# Increased Genomic Prediction Accuracy in Wheat Breeding Through Spatial Adjustment of Field Trial Data

**DOI:** 10.1534/g3.113.007807

**Published:** 2013-09-30

**Authors:** Bettina Lado, Ivan Matus, Alejandra Rodríguez, Luis Inostroza, Jesse Poland, François Belzile, Alejandro del Pozo, Martín Quincke, Marina Castro, Jarislav von Zitzewitz

**Affiliations:** * Programa Nacional de Investigación Cultivos de Secano, Instituto Nacional de Investigación Agropecuaria, Est. Exp. La Estanzuela, Colonia 70000, Uruguay; † Instituto de Investigaciones Agropecuarias, Centro Regional de Investigación Quilamapu, Casilla 426, Chillán, Chile; ‡ United States Department of Agriculture, Agricultural Research Service, Hard Winter Wheat Genetics Research Unit, Manhattan, Kansas 66506; § Department of Agronomy, Kansas State University, Manhattan, Kansas; ** Département de Phytologie and Institut de Biologie Intégrative et des Systèmes (IBIS), Université Laval, Québec, QC G1V 0A6, Canada; †† Universidad de Talca, Facultad de Ciencias Agrarias, Casilla 747, Talca, Chile

**Keywords:** genotyping-by-sequencing, genomic selection, wheat, single nucleotide polymorphism, quantitative trait locus, spatial Correction, GBLUP, Shared data resources, GenPred

## Abstract

In crop breeding, the interest of predicting the performance of candidate cultivars in the field has increased due to recent advances in molecular breeding technologies. However, the complexity of the wheat genome presents some challenges for applying new technologies in molecular marker identification with next-generation sequencing. We applied genotyping-by-sequencing, a recently developed method to identify single-nucleotide polymorphisms, in the genomes of 384 wheat (*Triticum aestivum*) genotypes that were field tested under three different water regimes in Mediterranean climatic conditions: rain-fed only, mild water stress, and fully irrigated. We identified 102,324 single-nucleotide polymorphisms in these genotypes, and the phenotypic data were used to train and test genomic selection models intended to predict yield, thousand-kernel weight, number of kernels per spike, and heading date. Phenotypic data showed marked spatial variation. Therefore, different models were tested to correct the trends observed in the field. A mixed-model using moving-means as a covariate was found to best fit the data. When we applied the genomic selection models, the accuracy of predicted traits increased with spatial adjustment. Multiple genomic selection models were tested, and a Gaussian kernel model was determined to give the highest accuracy. The best predictions between environments were obtained when data from different years were used to train the model. Our results confirm that genotyping-by-sequencing is an effective tool to obtain genome-wide information for crops with complex genomes, that these data are efficient for predicting traits, and that correction of spatial variation is a crucial ingredient to increase prediction accuracy in genomic selection models.

Wheat is the third most-important cereal crop in the world, with a total production of 704 million tons annually (FAOSTAT, 2011). To meet future market demands, some of the most important breeding objectives include increasing total yields and the rate at which wheat breeding programs adapt to new and changing environments.

New genomic tools in wheat breeding have allowed the incorporation of new allelic variants into adapted germplasm. Strategies like quantitative trait loci and association mapping have aided in identifying genes or genomic regions responsible for traits of interest (Lander and Botstein 1989; Jansen 1993; Tanksley 1993; Risch and Merikangas 1996; Pritchard *et al.* 2000; Kraakman *et al.* 2004; Kirigwi *et al.* 2007; Neumann *et al.* 2010; Le Gouis *et al.* 2012; Yu *et al.* 2012). Trait-associated markers then become selection targets to assist in molecular breeding programs (Collard *et al.* 2005; Landjeva *et al.* 2007; Collard and Mackill 2008; Buerstymayr *et al.* 2009). However, these approaches have limitations due to the difficulty in detecting significant markers within gene regions that are involved in the expression of complex traits influenced by many genes at different levels (Xu 2003). The most important traits involved in breeding are complex. Therefore, other strategies that take into account thousands of markers at one time in a model to predict complex traits have recently been developed.

Genomic selection (GS) is a recent approach that is being applied in crop breeding to make decisions for advancing germplasm from one generation to the next. GS was first proposed in animal breeding by Meuwissen *et al.* (2001). The development of high-throughput sequencing platforms, yielding a vast amount of information for each genotype allows the application of GS. For GS to be applicable in commercial breeding, genotyping methods need to be cost-effective. Genotyping-by-sequencing (GBS) is a high-throughput genotyping method that has been shown to be very useful for complex genomes like wheat (Poland *et al.* 2012a; Poland and Rife 2012). GBS costs are directly linked to the decreasing cost of sequencing driven by global research into developing new low-cost sequencing technologies and platforms. The wheat genome is very large at16 Gb (*i.e.*, five times the human genome) and very complex with 80% repeated regions and 25–30% of its genes duplicated (Bennett and Smith 1976; Dubcovsky *et al.* 1996; Akhunov *et al.* 2003). Furthermore, wheat is a hexaploid species with three genomes (A, B, and D) per chromosome (Sarkar and Stebbins 1956; Dvorak and Zhang 1990; Dvorak *et al.* 1993). GBS uses methylation sensitive enzymes, which results in the elimination of most of the repeated regions and reduces the genome representation to increase the efficiency of sequencing (Elshire *et al.* 2011; Sonah *et al.* 2013).

The development of novel statistical approaches for GS is a crucial step, where all the genotypic information is taken into account to be associated with phenotypic data by adjusting the parameters in a prediction model. The parameters of the model are currently being adjusted with linear models (Ridge regression [RR]), Bayesian approaches (Bayes A and Bayes B), and semiparametric strategies (reproducing Kernel Hilbert spaces [RKHS] and neural networks) (Meuwissen *et al.* 2001; Gianola *et al.* 2003, 2011; de los Campos *et al.* 2010; Endelman 2011; Goddard *et al.* 2011; Poland *et al.* 2012b). RKHS is defined by a “reproducing kernel,” which is a function of the relationship between pairs of genotypes. RKHS is a semiparametric approach, which could be represented as a parametric model by choosing the appropriate kernel (de los Campos *et al.* 2010). Two kernels that are commonly applied are known as RR and Gaussian (GAUSS). The relationships between genotypes in RR are established by the use of an additive model, and in GAUSS, the relationship between individuals is calculated with Euclidean distances that take into account epistatic interactions (Gianola and Van Kaam 2008; Endelman 2011).

Because genotyping is taking a more routine and accepted approach, improvements in model predictions are focused on precision phenotyping. Physiological differences and environmental conditions that affect the precision of the measured phenotype need to be taken into account to have an accurate GS prediction model. The breeder needs to know with accuracy the fields in which selections will occur; therefore, high-throughput phenotyping technologies are being implemented before planting (characterization of field heterogeneity) and during crop development to help reduce nongenetic variation (Crossa *et al.* 2006; Cabrera-Bosquet *et al.* 2012; Masuka *et al.* 2012; White *et al.* 2012). Otherwise, traditional methods are applied in which field design takes care of most of the variation and model correction mechanisms take into account the field heterogeneity that produces spatial correlation errors. These spatial trends can be eliminated with postdata treatment. Different strategies exist, including model variance-covariance matrixes, row-columns, and moving-means (Cullis *et al.* 1998; Peiris *et al.* 2008; Müller *et al.* 2010; Leiser *et al.* 2012).

The objectives of this study were (1) to validate the GBS technology as a tool for genotyping germplasm with complex genomes; and (2) to create an optimized training model for GS with a germplasm to be bred in a Mediterranean climate environment of central Chile, using a diverse set of wheat genotypes. Our study confirms that GBS is an inexpensive, robust, and useful tool to obtain genomewide information for breeding programs that work with complex genomes, such as wheat. Furthermore, we evaluate that spatial adjustment of the phenotypic data in each trial is very important to reduce error in the model and increase prediction accuracy. Here we evaluate spatial variation across the field, while also exploring fundamental variations that take into account environmental and genotypic interactions.

## Materials and Methods

### Germplasm and growth conditions

The germplasm consists of 384 advanced lines from two different breeding programs, including 55 lines from the wheat breeding program at Instituto Nacional de Investigación Agropecuaria (INIA) in Chile, 143 from the International Wheat and Maize Improvement Centre (CIMMYT) that were previously selected for adaptiveness to Chilean environments (these lines share common ancestors with the INIA-Chile breeding program), and 186 lines from INIA in Uruguay. The objective with this set of lines was to create a germplasm base to breed for drier areas in Chile and subsequently other countries within the projects involved.

The breeding germplasm was evaluated in the Mediterranean environment Santa Rosa (36°32′ S, 71°55′ W; 217 m.a.s.l.) under two levels of water supply, mild water stress and fully irrigated, in 2011. In 2012 the lines were evaluated in Santa Rosa under the two levels of water supply and also evaluated in Cauquenes (35° 58′ S; 72° 17′ W), a traditionally dry-land agricultural region with lower yield potential. Cauquenes has a granitic soil (Alfisol) with low fertility; the minimum average temperature is 4.7° (July), the maximum is 27° (January) and the long-term average annual precipitation is 695 mm. Santa Rosa has a volcanic soil (Andisol) with adequate fertility for wheat; the minimum average temperature is 3.0° (July), the maximum is 28.6° (January), and long-term average annual precipitation is 1270 mm (del Pozo and Del Canto 1999).

The experimental design was an alpha-lattice with 20 incomplete blocks, with each block containing 20 genotypes. Two replicates were used at both trials of Santa Rosa in 2011 and 2012 and at Cauquenes in 2012. Plots consisted of five rows of 2 m long and 0.2 m distance among rows. Sowing dates were on August 31st and September 7th at Santa Rosa and Cauquenes, respectively, and the sowing rate was 20 g m^2^. Plots were fertilized with 260 kg ha^−1^ of ammonium phosphate (46% P_2_O_5_ and 18% N), 90 kg ha^−1^ of potassium chloride (60% K_2_O), 200 kg ha^−1^ of sulpomag (22% K_2_O, 18% MgO, and 22% S), 10 kg ha^−1^ of boronatrocalcita (11% B), and 3 kg ha^−1^ of zinc sulfate (35% Zn). During tillering, an extra 80 kg ha^−1^ of N was applied. Weeds were controlled with MCPA at 750 g a.i. ha^−1^ + Metsulfuron Metil 8 g a.i. ha^−1^. Furrow irrigation was used at Santa Rosa: one irrigation (at tillering) for the mild water stress trial and four irrigations (at tillering, flag leaf emergence, heading, and middle grain filling) of ca. 50 mm each for the fully irrigated trial.

For phenotyping, all lines were evaluated for grain yield (GY), thousand kernel weight (TKW), number of kernels per spike (NKS), and days to heading (DH) in 2011. In 2012, only GY was evaluated. For the yield components (TKW and NKS), 25 spikes were randomly selected from each plot. For GY, the whole plot was harvested. DH was recorded as the number of days from sowing till 50% of spikes emerged.

### SNP identification

Genomic DNA was extracted using DNeasy Plant Maxi Kit (QIAGEN). Library construction was followed by using the *Pst*I-*Msp*I GBS protocol described by Poland *et al.* (2012a). This step and sequencing was performed twice. The libraries were made in collaboration with the Kansas State University, Manhattan, Kansas, and the Institut de Biologie Intégrative et des Systèmes at the Université Laval, Quebec, Canada. The sequencing was performed on an Illumina Hi-Sequation 2000 at the DNA core facility at the University of Missouri, Columbia, Missouri, and the McGill Univesity-Génome Quebec Innovation Centre (Montreal, Canada) for each set of libraries. The sequences were analyzed, in relation to base quality and distribution of sequence in different samples, using the Galaxy (http://galaxy.psu.edu/) software.

Single-nucleotide polymorphism (SNP) calls were made using the Tassel Pipeline (TP; http://maizegenetics.net), with modification for nonreference SNP calling by Poland *et al.* (2012a). The TP handles the sequence information coming from next-generation sequencing. Tags are defined, which are a set of identical sequences, and then the number of sequences per tag are counted. To handle tags with sequencing errors, the parameter to eliminate tags was established at less than 15 sequences. Tags are then defined individually by the lines that it came from. A pairwise alignment between tags to call some set of potential SNPs is then established. The TP has different filters for calling SNPs. In this study inbred lines that are in a highly homozygous state were used; therefore, the “inbreeding coefficient” filter was used and set to 0.9 to eliminate high amounts of heterozygotes. The minor allele frequency filter was set to 0.01, and the minimum locus coverage was set to eliminate SNPs with more than 80% missing data. Once the complete SNP matrix was established (supporting information, File S1), the missing data were imputed using the realized relationship matrix method multivariate normal expectation maximization (MVN-EM) described by Poland *et al.* (2012b) with the R environment (R Development Core Team 2008) package rrBLUP (Endelman 2011). To further verify that the imputed SNPs were not affected, we correlated the genetic relationship matrix with and without imputed SNPs. Then, to further verify the quality of the imputed SNPs, marker-based kinship matrixes between random subsets of SNPs were calculated and compared with the rrBLUP software package (Endelman 2011).

### Genetic and phylogenetic comparisons

SNP data from 384 diverse wheat genotypes were used to calculate the kinship (A) matrix of genotypes using the EMMA package (Kang *et al.* 2008) in R environment (R Development Core Team 2008). The dissimilarity matrix (1 − similarity matrix) was analyzed by principal coordinate analysis (PCoA) by use of the ape package (Paradis *et al.* 2004) in R.

### Sequence alignments

The SNP tags were BLASTed against the sequence database available from the Synthetic x Opata map by Poland *et al.* (2012a) (available at http://www.wheatgenetics.org/index.php/download/viewcategory/10-synop) using blastn from package NCBI-BLAST+ (Altschul *et al.* 1990) setting the parameters, maximum target, and number of threads at 1 and percent of identity at 95%.

Linkage disequilibrium (LD) between each pair of mapped SNPs was calculated as r^2 using the trio package (Schwender *et al.* 2012) in R. SNPs were ordered following the bin map order presented in the database used by Poland *et al.* (2012a). After SNPs were ordered, the LD values were plotted using the LDheatmap package (Shin *et al.* 2006) in R.

### Phenotypic predictions

**Figure 1:**
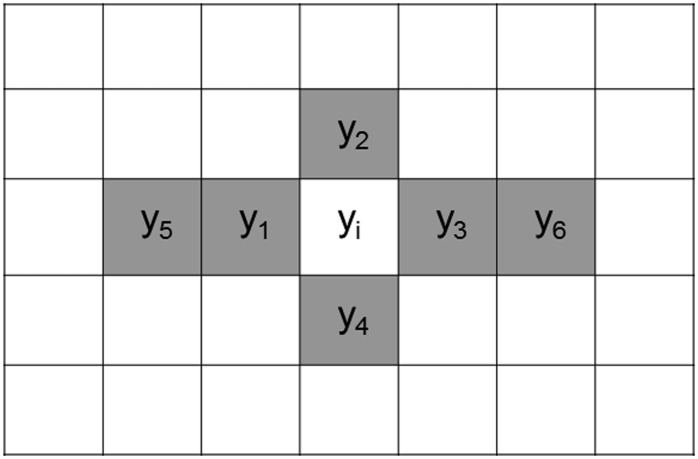
Diagram to calculate the covariable x_i_. Y_i_ is the phenotypic value in the plot. The neighboring plots are indicated with gray color.

Phenotypic data were analyzed using the lme4 (Bates 2007) and mvngGrAd (Technow 2012) packages in R. The analysis was performed individually for each condition and year. Two different mixed models and one linear regression model (Table 1), defined as Row-by-Column (RC), Random Complete Block model with moving means (RCB_MVNG), and linear regression model with moving means as covariable (MVNG), were considered to account for spatial correlations. Two of the four models use a covariable to correct for spatial variation in the field. The covariable (x_i_) was calculated as the value of phenotypic plot minus mean phenotypic value of neighbors plots, x_i_ = y_i_ – mean (y_1_, y_2_, y_3_, y_4_, y_5_, y_6_; Figure 1).

**Table 1:** Description of models used to adjust the phenotypic data

Model Name	Model Expression
IB	y = g _i_ + rep_j_ + bl(rep)_ijk_ + e_ijk_
RC	y = g _i_ +rep _j_ + fil(rep) _jk_ + col(rep) _jl_ + e_ijkl_
RCB_MVNG	y = g_i_ + βx_j_ + rep_k_ + e_ijk_
MVNG	y = u + βx_i_ + e_i_

IB, incomplete blocks, field design; g, treatment; rep, repetitions; bl(rep), incompletes blocks nested in repetitions; e, residual; u, general mean; RC, row by column model; fil(rep), rows nested in repetitions; col(rep), columns nested in repetitions; RCB_MVNG, random complete block model with moving means as covariable; x, covariable as phenotypic value of plot minus means of neighbors plots within grid; MVNG, linear regression model with moving means as covariable.

The three mixed models have a general expression as follows:

y=βX+Zu+e

where X is the design matrix for fixed effects β, Z is the design matrix for random effects, and *u* and *e* are the residual matrix that follows the distribution *e* ~ N(0,σ_e_^2^I). After the analysis of the residuals from each phenotypic model was established, the best linear unbiased predictors (BLUPs) were obtained to calculate genomic predictions by genomic best linear unbiased predictor (GBLUP) using the rrBLUP package with two different kernels, RR and GAUSS (Endelman 2011). The predictions were validated with 100 replications using the cross validation method described by Crossa *et al.* (2010). The samples were subdivided in seven similar sets. The training population was composed of six of the sets (86% of the samples) and the validation was performed on the remaining set (14% of the samples). For predictions between environments, adjusted data from two environments were used to train predictions in the remaining three environments.

## Results

### SNP identification

GBS SNPs were identified among sequences tag pairs by allowing one to three mismatches between tags. Two library replicates for the 384 samples were analyzed jointly for SNPs, producing a total of 102,324 SNPs. Similarity matrices calculated with and without imputation shown high correlation (r = 0.990, *p* < 0.001).

### Genetic and phylogenetic comparisons

**Figure 2:**
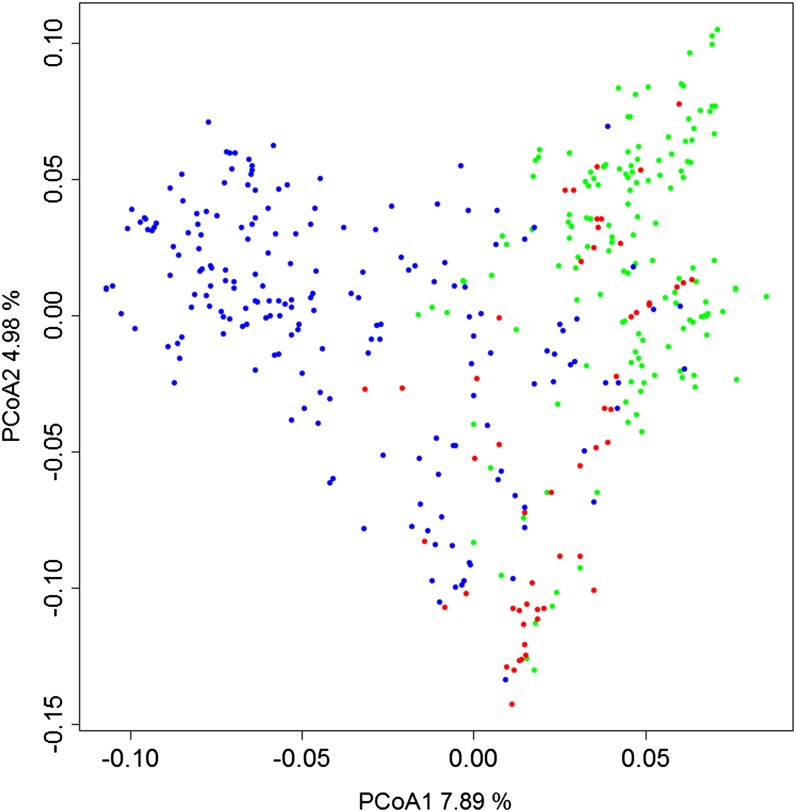
PCoA from dissimilarity matrix calculated with genetic data. Points in red represent advanced lines from the INIA Chile breeding program, green points identify lines from CIMMYT, and blue points denote advanced lines and prebreeding lines from the INIA Uruguay breeding program.

To test the predictability of the markers in constructing a genetic relationship matrix, the 102,324 SNPs were divided into two randomly assigned identical sets of 51,162 SNPs in each group. Two genetic similarity matrices were constructed independently with each of the sets of SNPs. The Pearson correlation between matrices was 0.997 (*p* = 0.001). A genetic similarity matrix was calculated with the complete set of markers to perform a principal coordinate analysis. The germplasm was separated in two groups, representing each breeding program (CIMMYT-INIA Chile and INIA Uruguay). The first two principal coordinates explained 12.9% of variation (Figure 2).

### Sequence alignments

**Figure 3:**
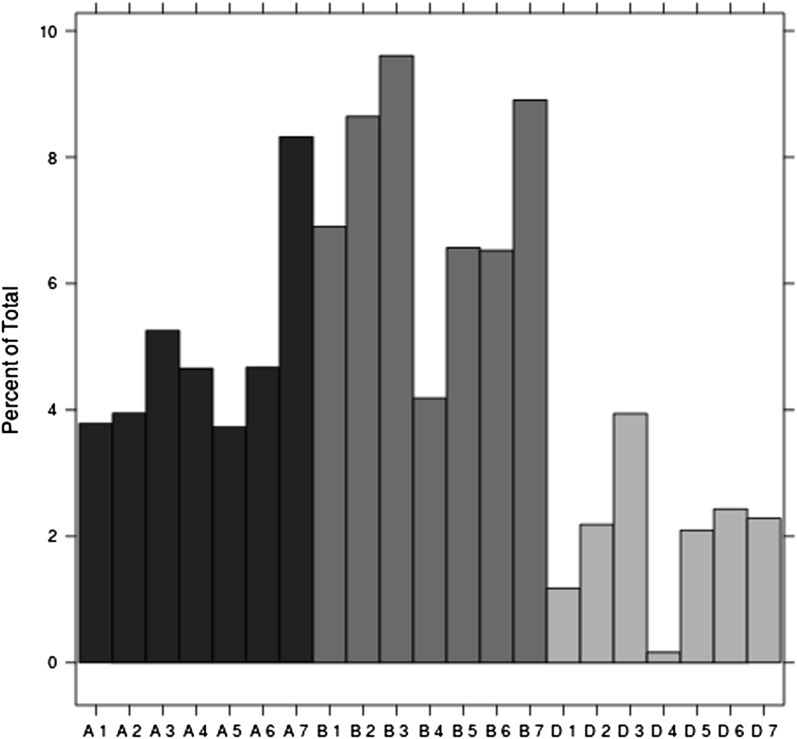
GBS-based SNP distribution along different wheat chromosomes.

When comparing sequences using BLAST against the Poland *et al.* (2012a) GBS-based SNP database, we found that the sequences in common showed a good coverage throughout all 21wheat linkage groups. Of all the SNPs, 13% (13,357) found high-quality matches. Although a good coverage was observed, the D genome presented fewer SNPs than the A and B genomes (Figure 3). As expected, LD analysis between mapped SNPs indicated high LD between closely linked SNPs along all chromosomes (File S1).

### Phenotypic analysis

**Figure 4:**
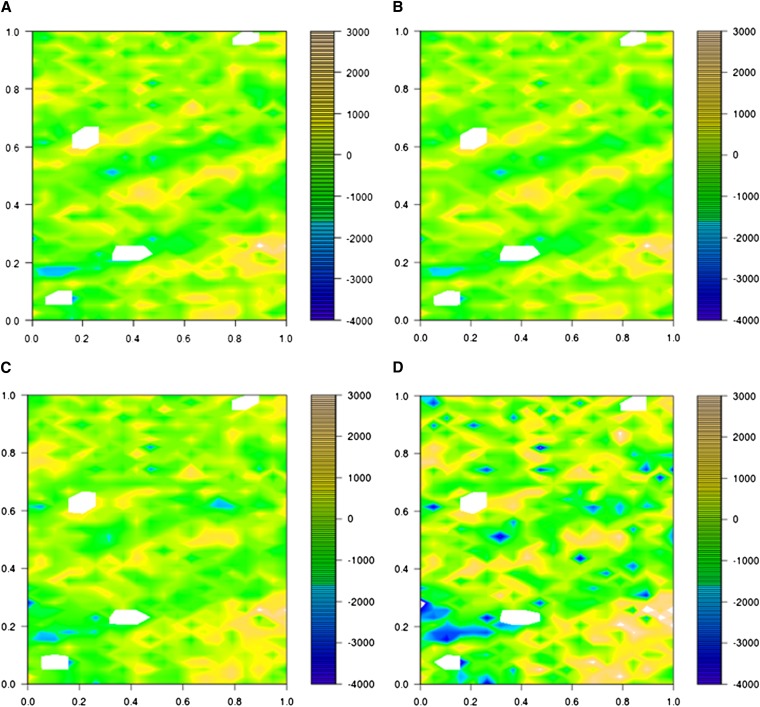
Plot residuals along the field for each model analysis for Santa Rosa irrigated. The color scale shows the value of residuals as indicated. (A) Residuals for incomplete blocks, field design; (B) residuals for RC; (C) residuals for RCB_MVNG; (D) residuals for MVNG.

**Figure 5:**
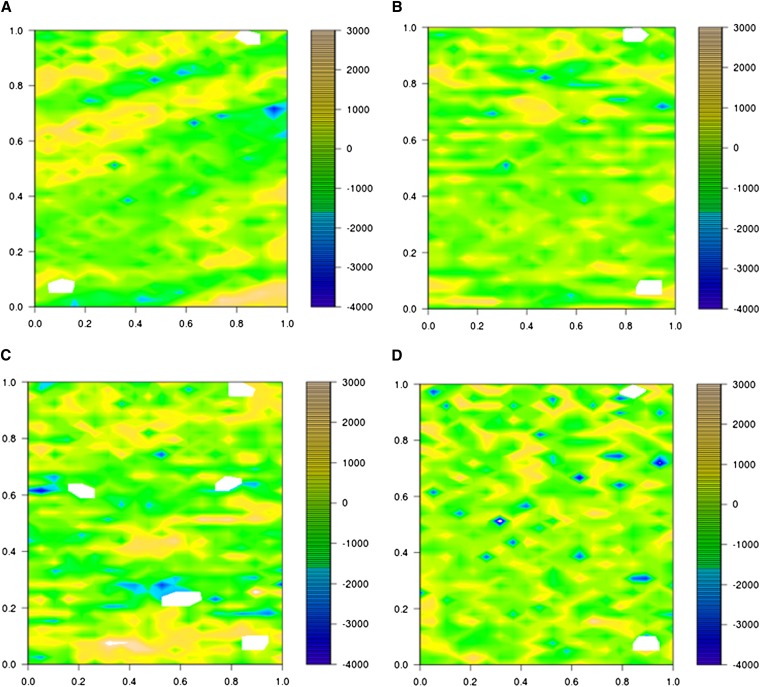
Plot residuals along the field for each model analysis for Santa Rosa nonirrigated trial. The color scale shows the value of residual effects as indicated. (A) Residuals for incomplete blocks, field design; (B) residuals for RC; (C) residuals for RCB_MVNG; (D) residuals for MVNG

Phenotypic data were collected under different soil water availability in 2011 and 2012. The traits under study (GY, TKW, NKS, DH) were analyzed adjusting for field design and for spatial variation using linear mixed models. The residuals for the adjusted traits in 2011 were heterogeneous due to spatial correlations (Figure 4 and Figure 5). Other models (RC, RCB_MVNG, and MVNG) were considered to reduce correlations between residuals. The RC model was inadequate to correct the residual heterogeneity, because the same spatial correlation was observed (Figure 4 and Figure 5). The other two (RCB_MVNG and MVNG) models adjusted, which include the moving means as a covariable, presented homogeneous residuals along the field (Figure 4 and Figure 5). In addition, the broad sense heritability was calculated for each model. The greatest heritability value was for the MVNG model for all traits (Table 2).

**Table 2:** Broad sense heritability for each field trial

		2011			2012
		H^2^ IB	H^2^ RC	H^2^ RCB_MVNG / MVNG	H^2^
SR_FI	GY	0.42	0.44	0.57	0.622
	TKW	0.88	0.88	0.89	—
	DH	0.95	0.95	0.95	—
	NKS	0.76	0.76	0.74	—
SR_MWS	GY	0.33	0.37	0.56	0.641
	TKW	0.79	0.81	0.82	—
	DH	0.93	0.93	0.93	—
	NKS	0.74	0.75	0.75	—
C_WS	GY	—	—	—	0.340

H^2^, broad sense heritability; IB, incomplete blocks, field design; RC, row by column model; RCB_MVNG, random complete block model with moving means as covariable; MVNG, linear regression model with moving means as covariable; SR_FI, Santa Rosa under Full Irrigation; SR_MWS, Santa Rosa under Mild Water Stress; C_SWS, Cauquenes under severe water stress; GY, grain yield; TKW, thousand kernel weight; DH, days to heading; NKS, number of kernels per spike.

In 2012 the fields had minimal spatial variation; therefore, after we adjusted the phenotypic data with the intended field design, the residuals we observed were homogeneous throughout the field. The heritability for yield was greater for the 2012 trials than for the 2011 trials. Cauquenes, which was a dry-land condition with more drought stress, presented a more pronounced field variation (Table 2) resulting in a lower heritability than the other two fields in 2012.

### Phenotypic predictions

A GS model using the GBLUP approach was fitted for each trait, using the line BLUPs of the best-fit phenotypic model. In all cases the 86% of genotypes were used to train the genomic model and the predictions were assessed in the other 14%. The predictions were evaluated using 100 cross validations with the training and prediction sets randomly partitioned.

We determined the standard deviation across all correlation for each model. The RR and GAUSS kernels were tested for each GS model. In most of the cases GAUSS performed better than RR. In general, the phenotypic data adjusted with MVNG, resulted in greater prediction accuracies, although high standard errors were observed (Table 3). The prediction accuracies for 2012 were greater than for 2011 (Table 3 and Table 4).

**Table 3:** Accuracy of predictions for each trial in 2011 using random training sets with 100 independent randomizations

			IB	RC	RCB_MVNG	MVNG
SR_FI	GY	RR	0.298 ± 0.117	0.296 ± 0.119	0.319 ± 0.114	0.319 ± 0.113
		GAUSS	0.312 ± 0.117	0.310 ± 0.120	0.325 ± 0.117	0.326 ± 0.116
	TKW	RR	0.780 ± 0.056	0.780 ± 0.056	0.777 ± 0.057	0.843 ± 0.040
		GAUSS	0.786 ± 0.055	0.786 ± 0.055	0.782 ± 0.056	0.847 ± 0.039
	DH	RR	0.409 ± 0.109	0.409 ± 0.109	0.405 ± 0.109	0.579 ± 0.123
		GAUSS	0.436 ± 0.111	0.436 ± 0.111	0.433 ± 0.111	0.614 ± 0.121
	NKS	RR	0.479 ± 0.114	0.479 ± 0.114	0.484 ± 0.115	0.665 ± 0.077
		GAUSS	0.487 ± 0.119	0.487 ± 0.119	0.492 ± 0.120	0.669 ± 0.075
SR_MWS	GY	RR	0.236 ± 0.141	0.275 ± 0.147	0.231 ± 0.127	0.347 ± 0.134
		GAUSS	0.231 ± 0.144	0.273 ± 0.150	0.260 ± 0.128	0.370 ± 0.132
	TKW	RR	0.759 ± 0.061	0.762 ± 0.061	0.757 ± 0.058	0.841 ± 0.034
		GAUSS	0.764 ± 0.059	0.767 ± 0.059	0.761 ± 0.057	0.845 ± 0.034
	DH	RR	0.398 ± 0.110	0.399 ± 0.110	0.396 ± 0.110	0.563 ± 0.134
		GAUSS	0.423 ± 0.108	0.423 ± 0.108	0.423 ± 0.108	0.604 ± 0.134
	NKS	RR	0.464 ± 0.115	0.466 ± 0.114	0.458 ± 0.114	0.608 ± 0.088
		GAUSS	0.483 ± 0.111	0.485 ± 0.111	0.478 ± 0.111	0.608 ± 0.086

IB, incomplete blocks, field design; RC, row by column model; RCB_MVNG, random complete block model with moving means as covariable; MVNG, linear regression model with moving means as covariable; SR_FI, Santa Rosa under full irrigation; GY, grain yield; RR, Ridge regression kernel; GAUSS, Gaussian kernel; TKW, thousand kernel weight; DH, days to heading; NKS, number of kernels per spike; SR_MWS, Santa Rosa under mild water stress.

**Table 4:** Accuracy of prediction for yield in 2012 using random training sets with 100 independent randomizations

	RR	GAUSS
SR_FI	0.487 ± 0.093	0.516 ± 0.086
SR_MWS	0.617 ± 0.078	0.626 ± 0.077
C_SWS	0.382 ± 0.104	0.378 ± 0.104

RR, Ridge regression kernel; GAUSS: Gaussian kernel; SR_FI, Santa Rosa under Full Irrigation; SR_MWS, Santa Rosa under Mild Water Stress; C_SWS, Cauqenes under Severe Water Stress.

All possible combinations of the trials were used to test as training sets to observe the prediction accuracy from year to year or from one environment to the next. Because of the high variation from year to year, the best predictions were obtained when adding information in the model that included both years (Table 5).

**Table 5:** Accuracy of predictions between different environments

	SR_FI2011		SR_MWS2011		C_SWS2012		SR_FI2012		SR_MWS2012	
1	0.292	0.319	0.263	0.294	0.414	0.405	−	−	−	−
2	0.221	0.234	0.192	0.205	−	−	0.626	0.637	−	−
3	0.291	0.312	0.251	0.275	−	−	−	−	0.761	0.760
4	0.569	0.641	−	−	0.319	0.310	0.592	0.624	−	−
5	0.626	0.681	−	−	0.258	0.249	−	−	0.622	0.619
6	0.628	0.718	−	−	−	−	0.403	0.426	0.458	0.453
7	−	−	0.560	0.639	0.329	0.326	0.592	0.620	−	−
8	−	−	0.604	0.662	0.271	0.269	−	−	0.610	0.615
9	−	−	0.624	0.693	−	−	0.430	0.445	0.466	0.465
10	−	−	−	−	0.088	0.109	0.330	0.358	0.303	0.325

In each case (1−10), two environments were used to train the prediction model. SR_FI2011, Santa Rosa Full irrigated in 2011; SR_MWS2011, Santa Rosa mild water stress in 2011; C_SWS2012, Cauqenes severe water stress in 2012; SR_FI2012, Santa Rosa full irrigated in 2012; SR_MWS2012,Santa Rosa mild water stress 2012.

The training sets were 1: SR_FI2012/SR_MWS2012; 2: C_SWS2012/SR_MWS2012; 3: C_SWS2012/SR_FI2012; 4: SR_MWS2011/ SR_MWS2012; 5: SR_MWS2011/SR_FI2012; 6: SR_MWS2011/C_SWS2012; 7: SR_FI2011/SR_MWS2012, 8: SR_FI2011/SR_FI2012; 9: SR_FI2011/C_SWS2012; 10: SR_FI2011/SR_MWS2011.

## Discussion

The challenge in wheat breeding is to accelerate the adaptation of germplasm for more efficient and rapid results, if possible, to increase yield, and to adapt to future climate change. Tools are now available that allow taking up this challenge and relate the complex genetic mechanism involved in phenotypic expression in relation with environmental interactions (Heffner *et al.* 2009).

### Genotypic analysis

We detected 102,324 SNPs using two library replicates, which were used to calculate a dissimilarity matrix, which was analyzed by PCoA. As described by Poland *et al.* (2012a), we didn’t expect big differences between dissimilarity matrixes calculates with and without imputation. For PCoA, the lines were grouped by breeding program of origin. This was expected as the lines from the same breeding program should be more similar genetically because they share a common parental background and were bred under similar developmental conditions (Rauf *et al.* 2010).

When BLASTing against tags in the SynOpDH genetic map, we found that 13% of the tags aligned with high similarity. These tags were used to test LD between SNPs (File S1). High LD groups were identified, which is expected if the genotypes in each sample are true calls. In the same way, the distribution and concentration of SNPs in different chromosomes was in agreement with other assessments of LD in elite wheat breeding lines (Chao *et al.* 2010). Although 13% does not seem like a high proportion, the resulting number of SNPs is 13,357, which are well distributed and is a high number of SNPs, allowing us to calculate LD across individuals and confirming high correlations between closely linked SNPs per linkage group. This evidence allows us to trust the quality of the SNPs identified through GBS for genomic predictions.

### Phenotypic predictions

Before predictions were made, the phenotypic data were adjusted and residuals in the model were analyzed. The analysis showed a strong spatial effect across the field for the 2011 data. The introduction of a spatial correction model enabled a better adjustment in measurements, showing an increment in the heritability of measurements. A greater heritability indicates that a greater proportion of the variance in the experiment is due to a difference between genotypes (Holland *et al.* 2003). The models that showed a greater heritability also showed an increase in the mean accuracies of genomic predictions. The importance of taking into account appropriate field design prior to the experiment has been demonstrated before (Federer 2003; Federer and Crossa 2012). Strategies for data analysis also have been described in cases in which the variability in the field has not been measured before applying the field design and has made the field design ineffective (Cullis *et al.* 1998; Piepho and Williams 2010). In this study we found that these postdata adjustments improved the quality of our data. As genotyping becomes less and less of a constraint for developing genomic prediction training sets, proper treatment of phenotypic observations is the key to increasing the accuracy of predictions. There are studies that include sets of environmental variables in training models, which are measured during experiments, with the objective to control these sources of variation and consider genotypic × environmental interactions (Chapman 2008). If the different sources of variation could be measured, together with genotypic × environment interactions, prediction accuracies should improve.

Another interest is the ability to predict phenotypes across environments, or from year to year (Burgueño *et al.* 2012). However, when predicting new lines in previously untested environments, most prediction power is lost. Therefore, it is important to generate a growing database for model training, with corresponding sets of genotypes for the breeding program and target environments and continually increase the number of environments tested. In the present study, greater prediction accuracies were observed when we used data from 2 years to train the model (Table 5). When training the model with data from only one year, the accuracies of predictions were low because the year-to-year correlation was low (Table 5).

The accuracies of predictions were comparable with other work in wheat, which have used different fingerprinting approaches (Crossa *et al.* 2010; Heffner *et al.* 2011), adding confidence to the GBS approach.

Two different statistical models were tested using the GBLUP approach. The greatest prediction accuracies were achieved with a Gaussian kernel. This model considers epistatic effects in addition to additive effects modeled presented in RR models (Gianola and Van Kaam 2008).

This study is part of a long-term objective to adapt wheat germplasm to Mediterranean climate environments of central Chile and subsequently to other regions in South America that are in similar needs. Previous data (I. Matus, unpublished data) and experience suggest that the 384 lines present traits incorporated from CIMMYT, show adaptation to drier areas in Chile, and that both germplasm groups (INIA-Chile and INIA-Uruguay) have shown different degrees of adaptation to these Chilean environments. We believe that this dataset contains the necessary genetic diversity for a germplasm base to start a breeding program guided toward drier areas in Chile.

The development of new genotyping tools has been the framework to the practical implementation of GS. There are many crops with different genomic characteristics that should be considered when identifying the most suited genotyping methodology. In this study, GBS was successfully applied for the genome-wide characterization of wheat breeding lines. GBS is a low-cost approach that can be used to genotype thousands of lines per year in a commercial breeding program.

The challenge in leveraging genomic assisted breeding approaches in applied programs now remains in obtaining high-quality and long-term accumulation of phenotypic data from multiple years and targeted environments. This study showed an increase in prediction accuracy with proper treatment of phenotypic data from field trials. High-throughput and high-precision phenotyping tools are being tested and used that will be well suited for breeding program and increasing predictions. Understanding and predicting the complex interaction between genotype and environment will also be key to select lines based on genotypic information.

## Supplementary Material

Supporting Information
