# Supplementary material for: Increased Genomic Prediction Accuracy in Wheat Breeding Through Spatial Adjustment of Field Trial Data
Source: G3 (Bethesda). 2013 Sep 30;3(12):2105–14. doi: 10.1534/g3.113.007807 (PMC3852373; doi:10.1534/g3.113.007807)
Supplement: Supporting Information [file supp_3_12_2105__index.html]

Increased Genomic Prediction Accuracy in Wheat Breeding Through Spatial Adjustment of Field Trial Data — Supporting Information 

# Increased Genomic Prediction Accuracy in Wheat Breeding Through Spatial Adjustment of Field Trial Data

## Supporting Information for Lado *et al.*, 2013

**Files in this Data Supplement:**

- File S1 - Phenotype data from 2011 and 2012, genotype data, and supporting figures (.zip, 15 MB)
