# Supplementary material for: Increased Genomic Prediction Accuracy in Wheat Breeding Through Spatial Adjustment of Field Trial Data
Source: G3 (Bethesda). 2013 Sep 30;3(12):2105–14. doi: 10.1534/g3.113.007807 (PMC3852373; doi:10.1534/g3.113.007807)
Supplement: Supporting Information [file supp_g3.113.007807_FileS1.zip › FileS1/Supplement1.pdf]

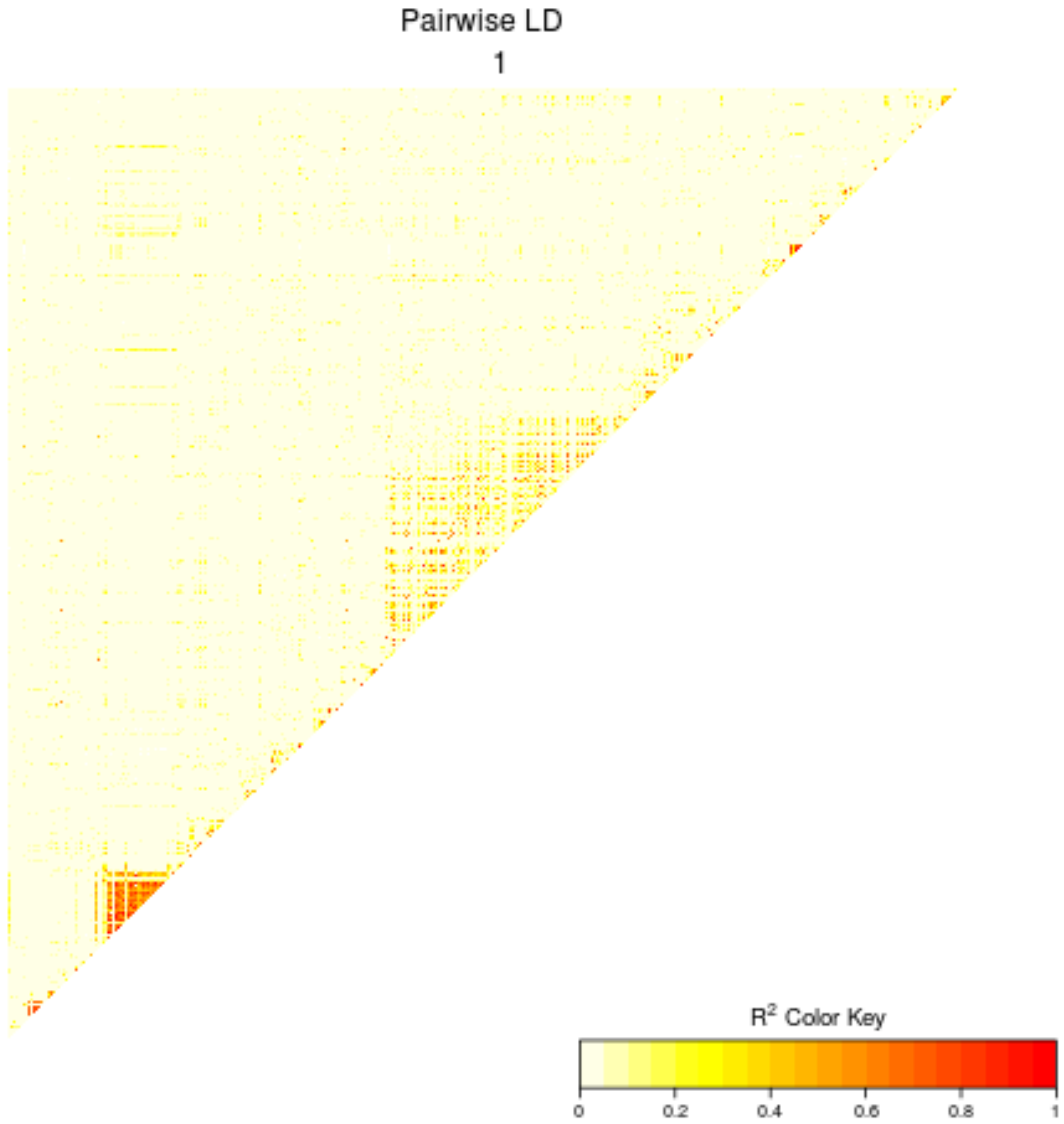

Pairwise LD
1
R^2 Color Key
0
0.2
0.4
0.6
0.8
1

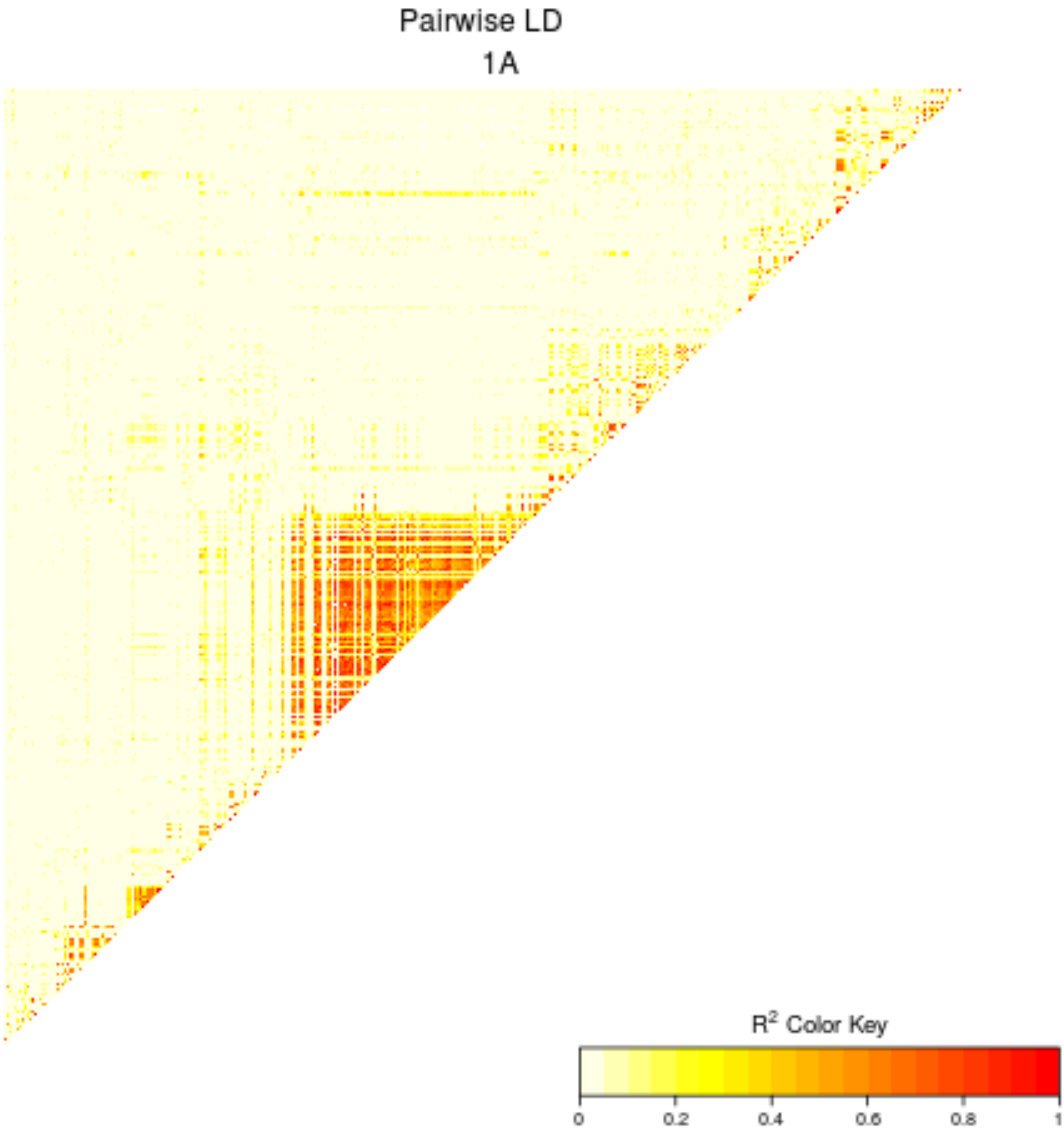

Pairwise LD
1A
R2 Color Key
0
0.2
0.4
0.6
0.8
1

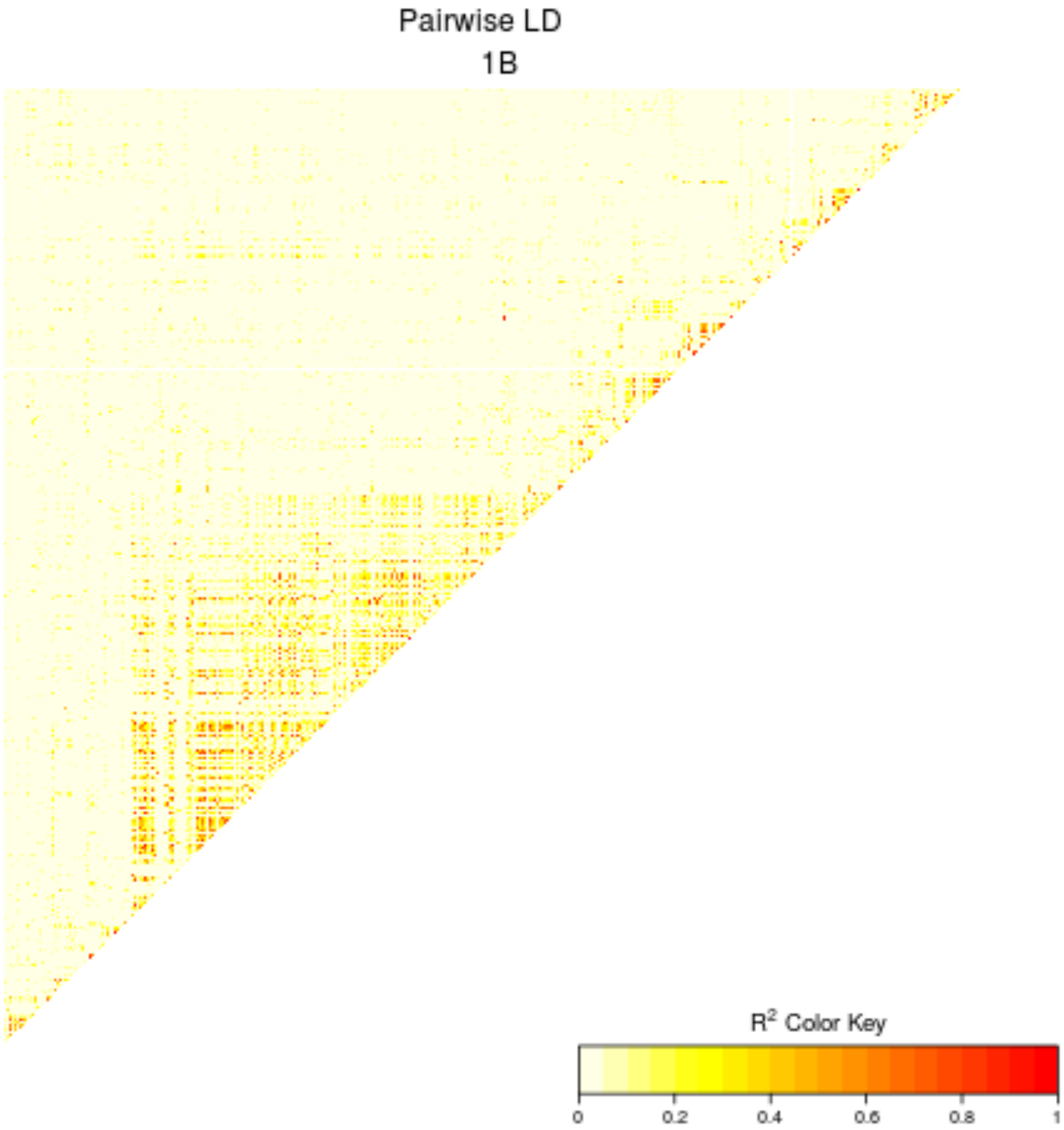
Pairwise LD
1B
R2 Color Key
0
0.2
0.4
0.6
0.8
1

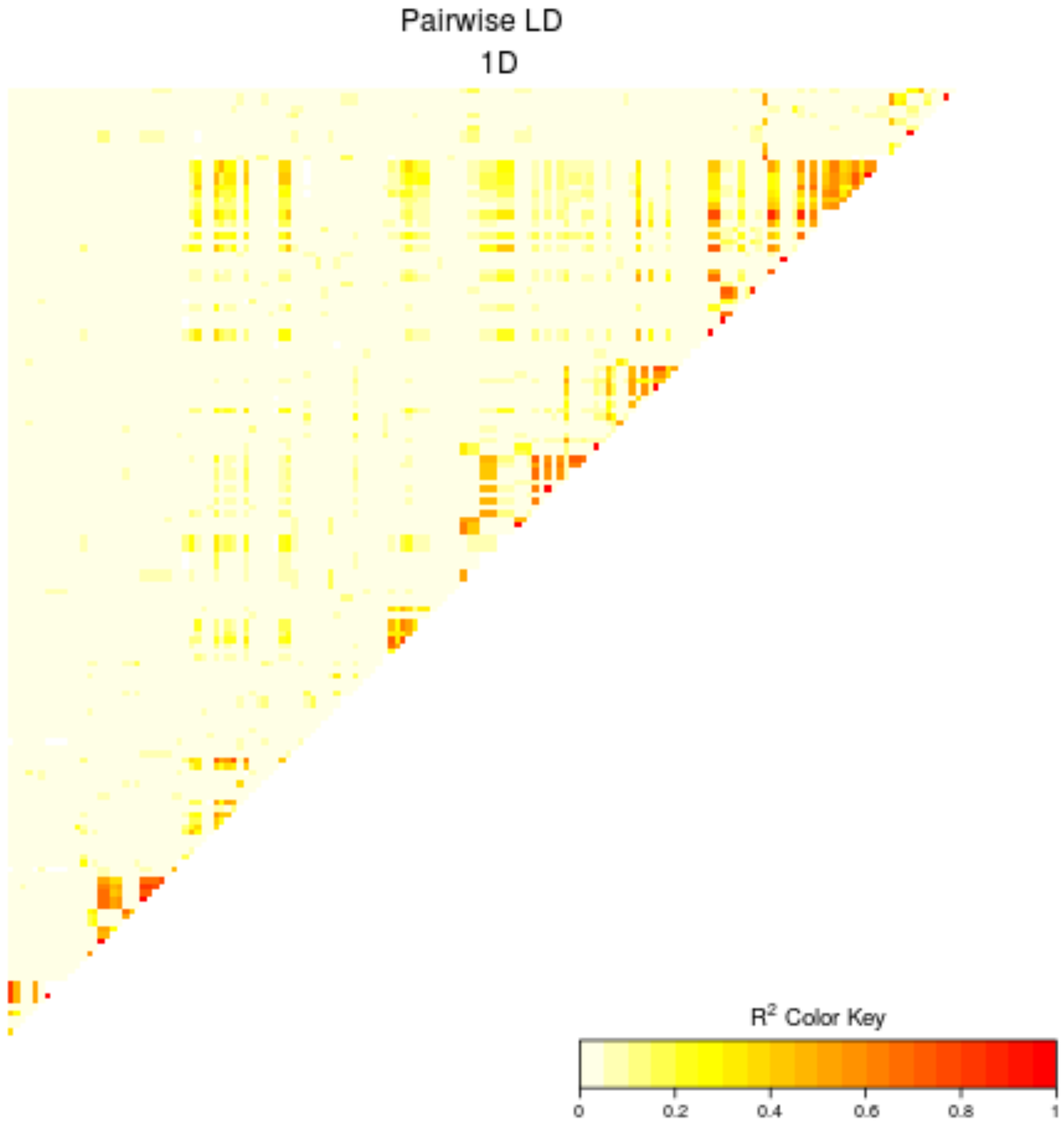

Pairwise LD
1D
R2 Color Key
0
0.2
0.4
0.6
0.8
1

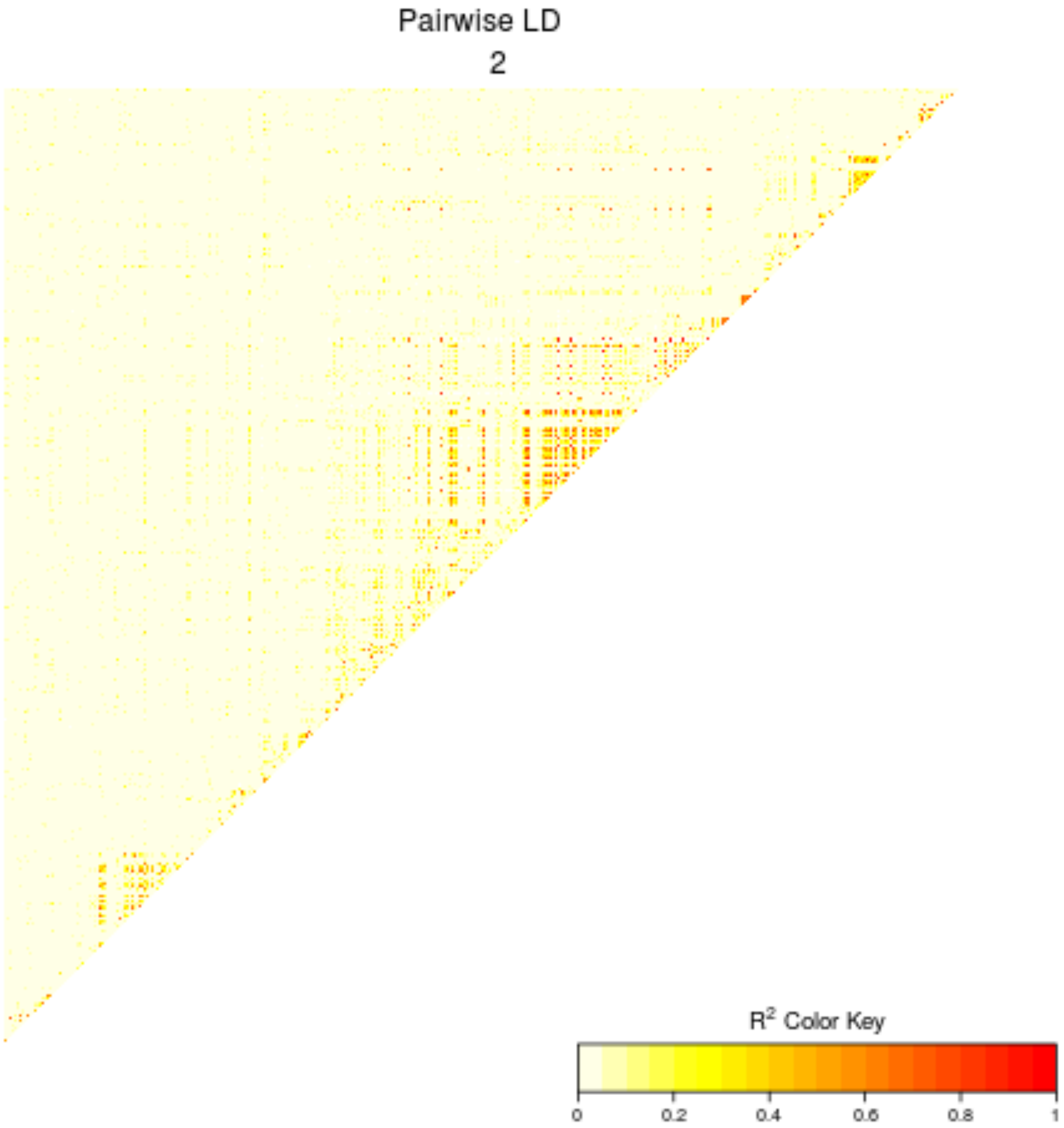
Pairwise LD
2
R² Color Key
0
0.2
0.4
0.6
0.8
1

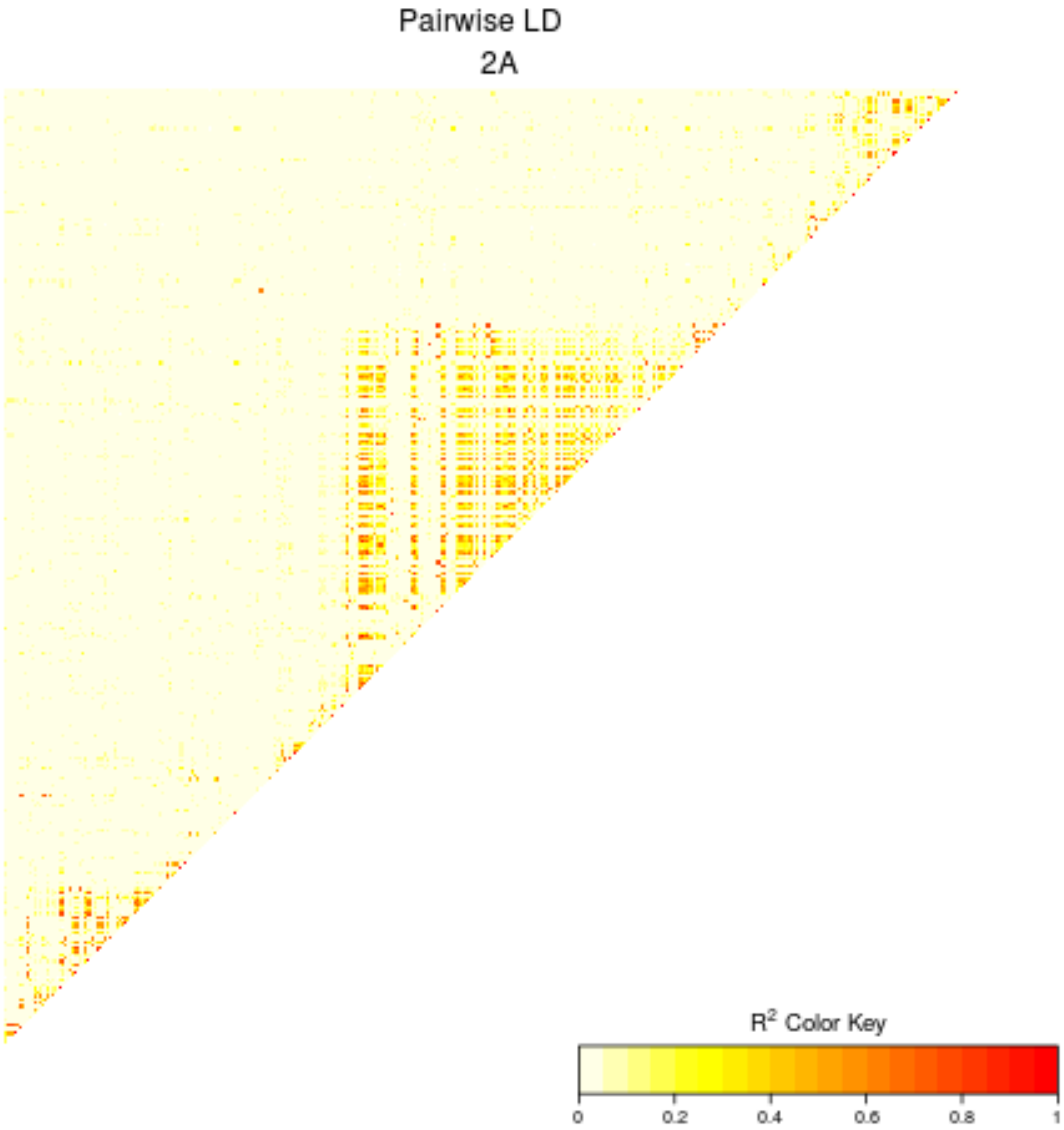
Pairwise LD
2A
R2 Color Key
0
0.2
0.4
0.6
0.8
1

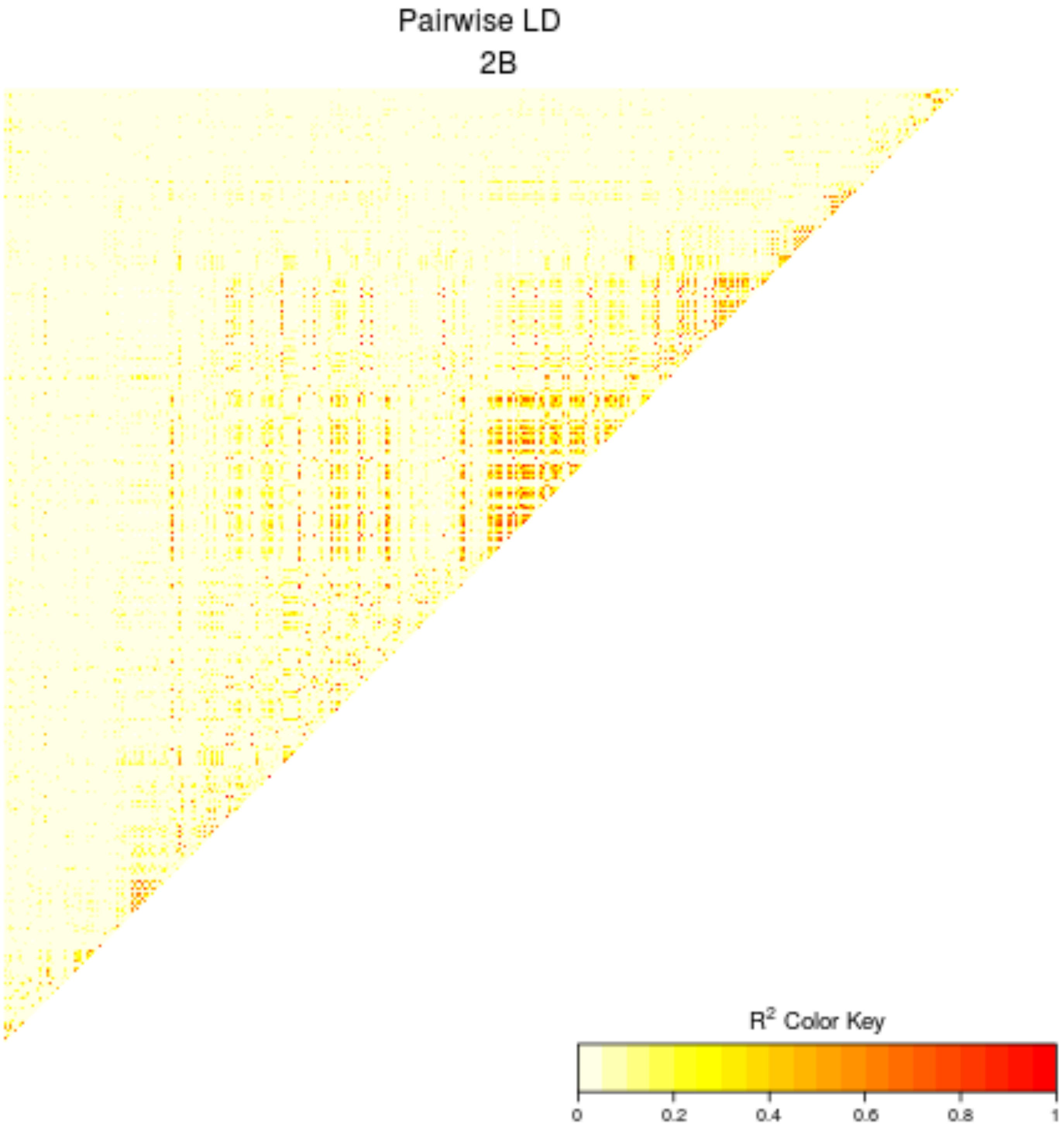
Pairwise LD
2B
R2 Color Key
0
0.2
0.4
0.6
0.8
1

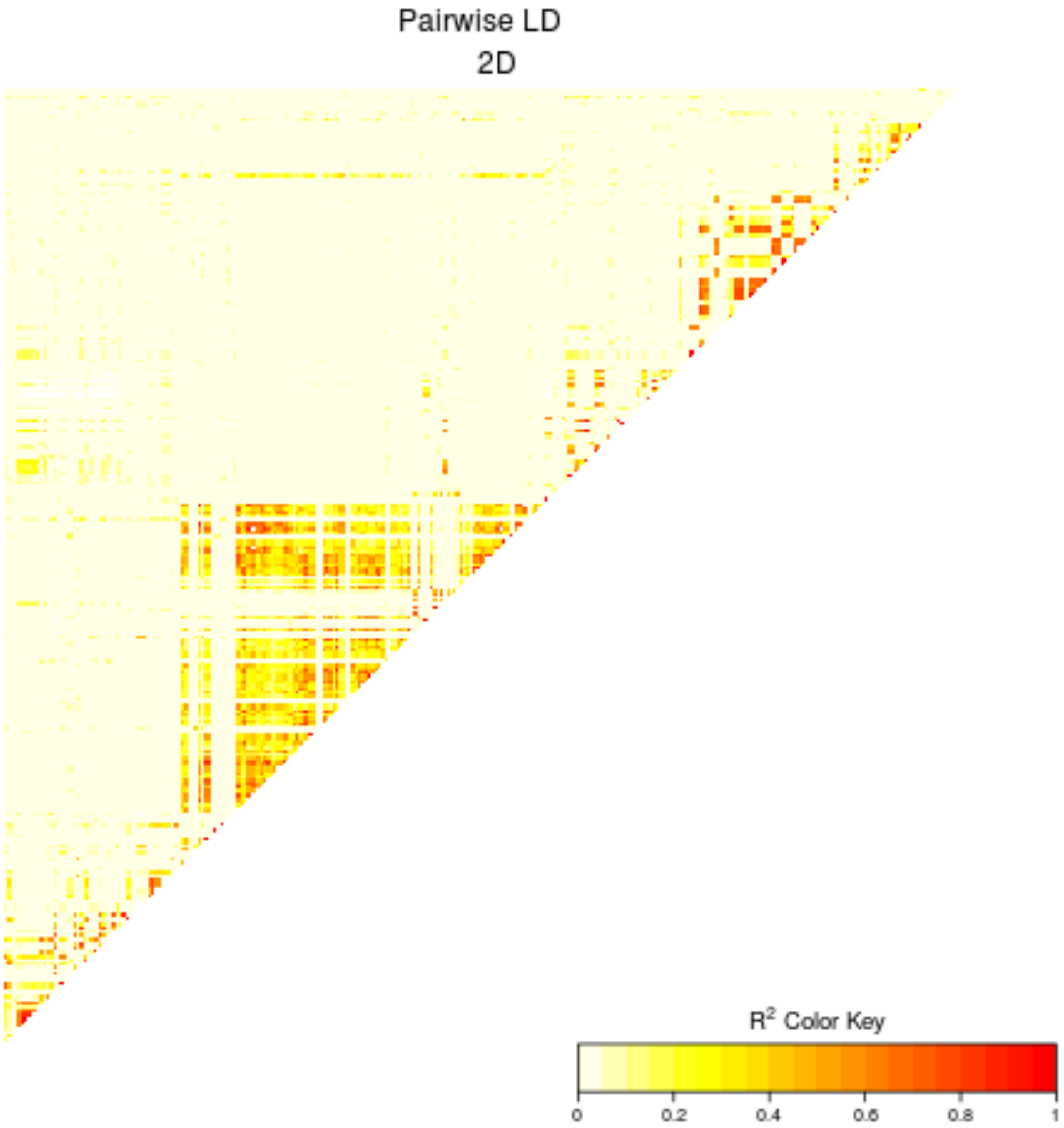

Pairwise LD
2D
R2 Color Key
0
0.2
0.4
0.6
0.8
1

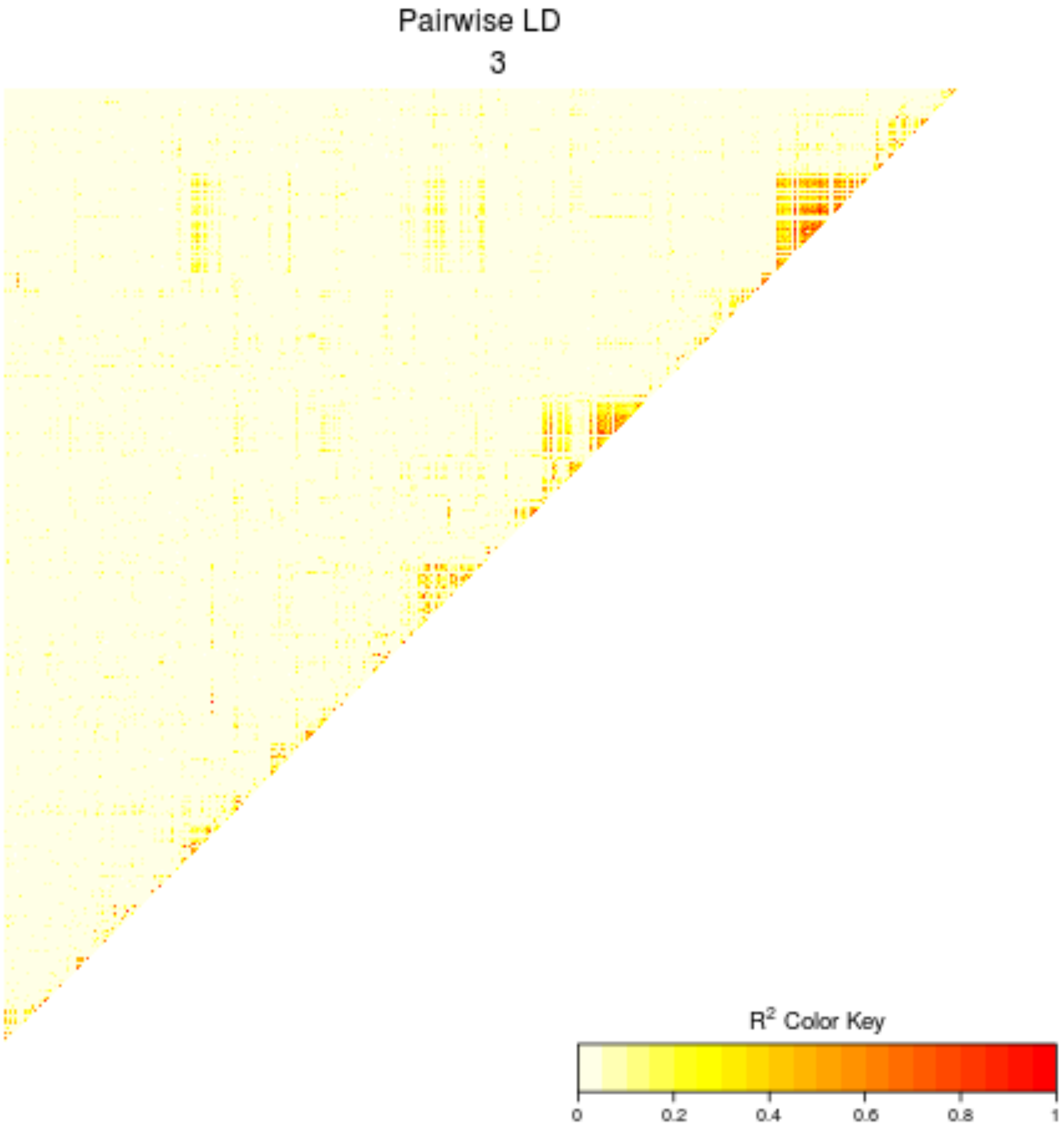
Pairwise LD
3
R2 Color Key
0
0.2
0.4
0.6
0.8
1

Pairwise LD
3A

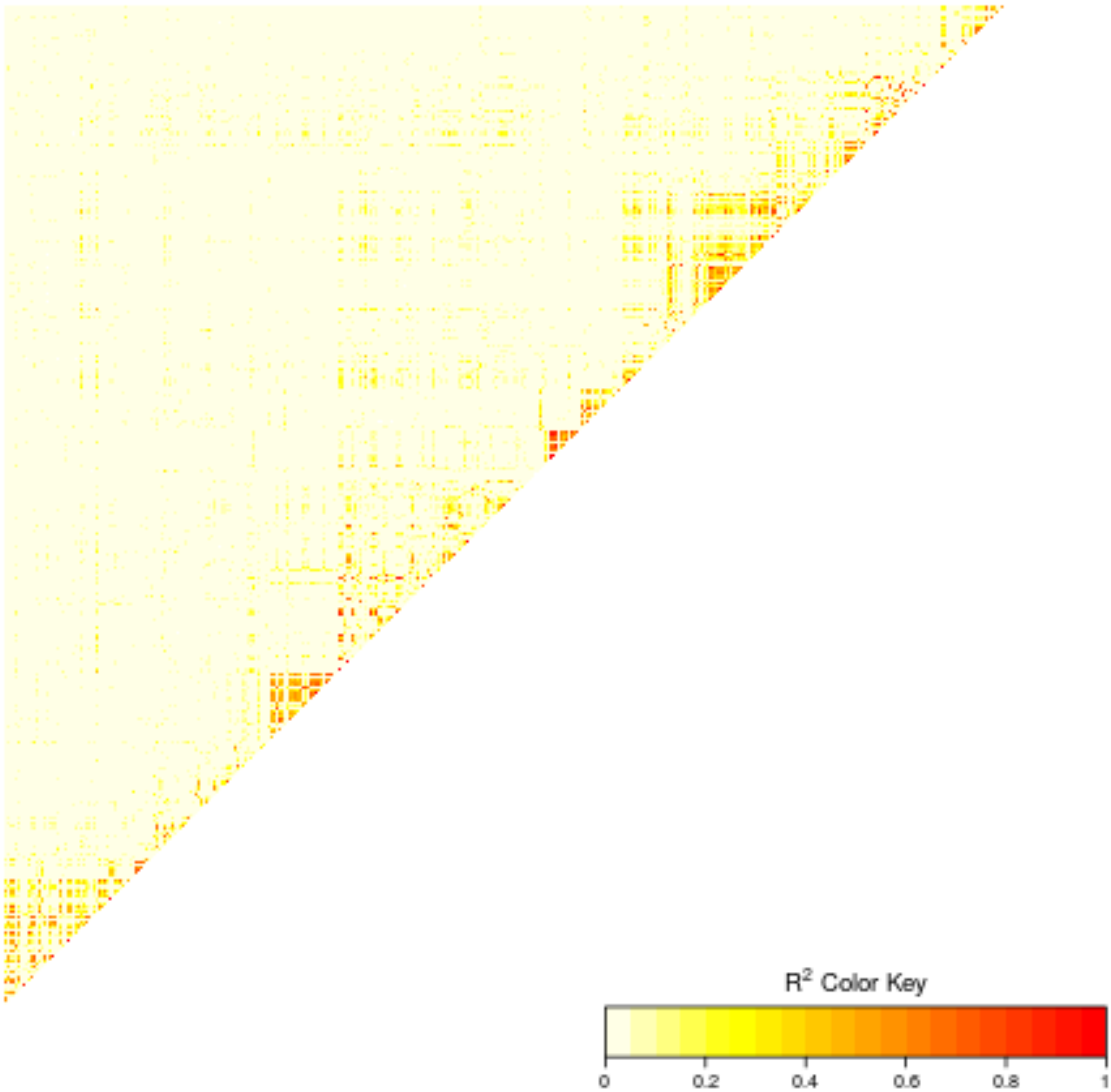

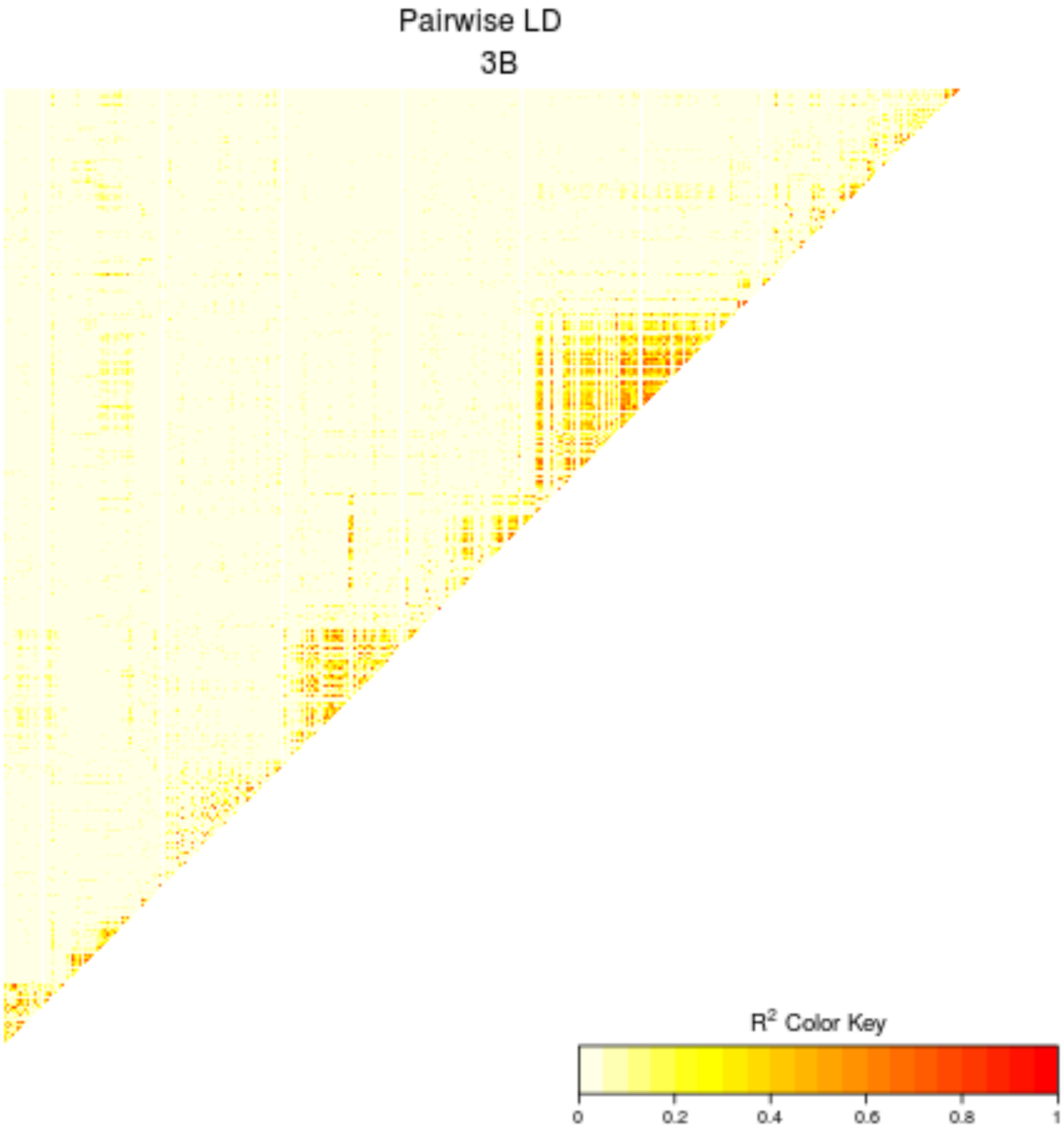
Pairwise LD
3B
R2 Color Key
0
0.2
0.4
0.6
0.8
1

Pairwise LD
3D

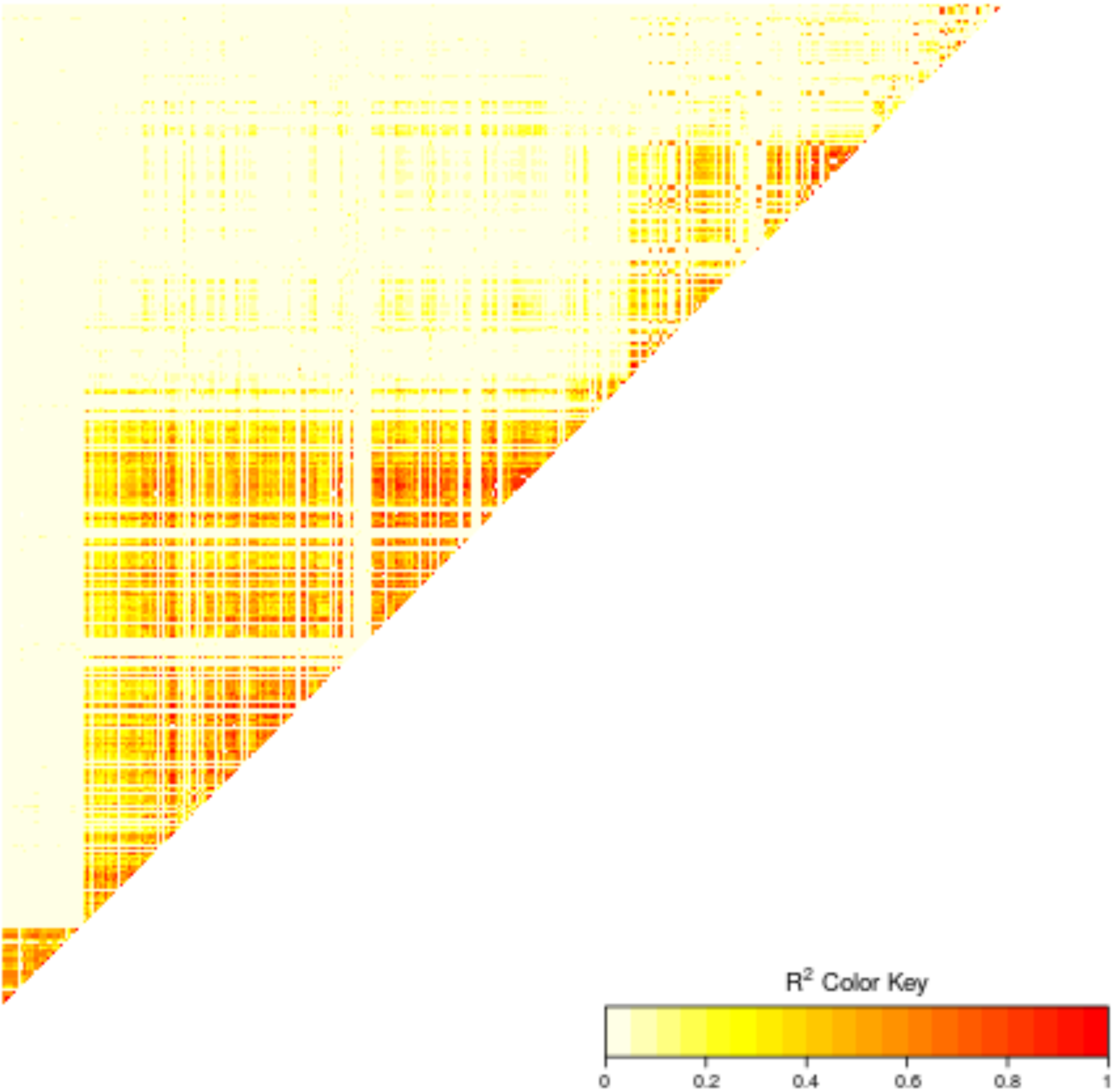

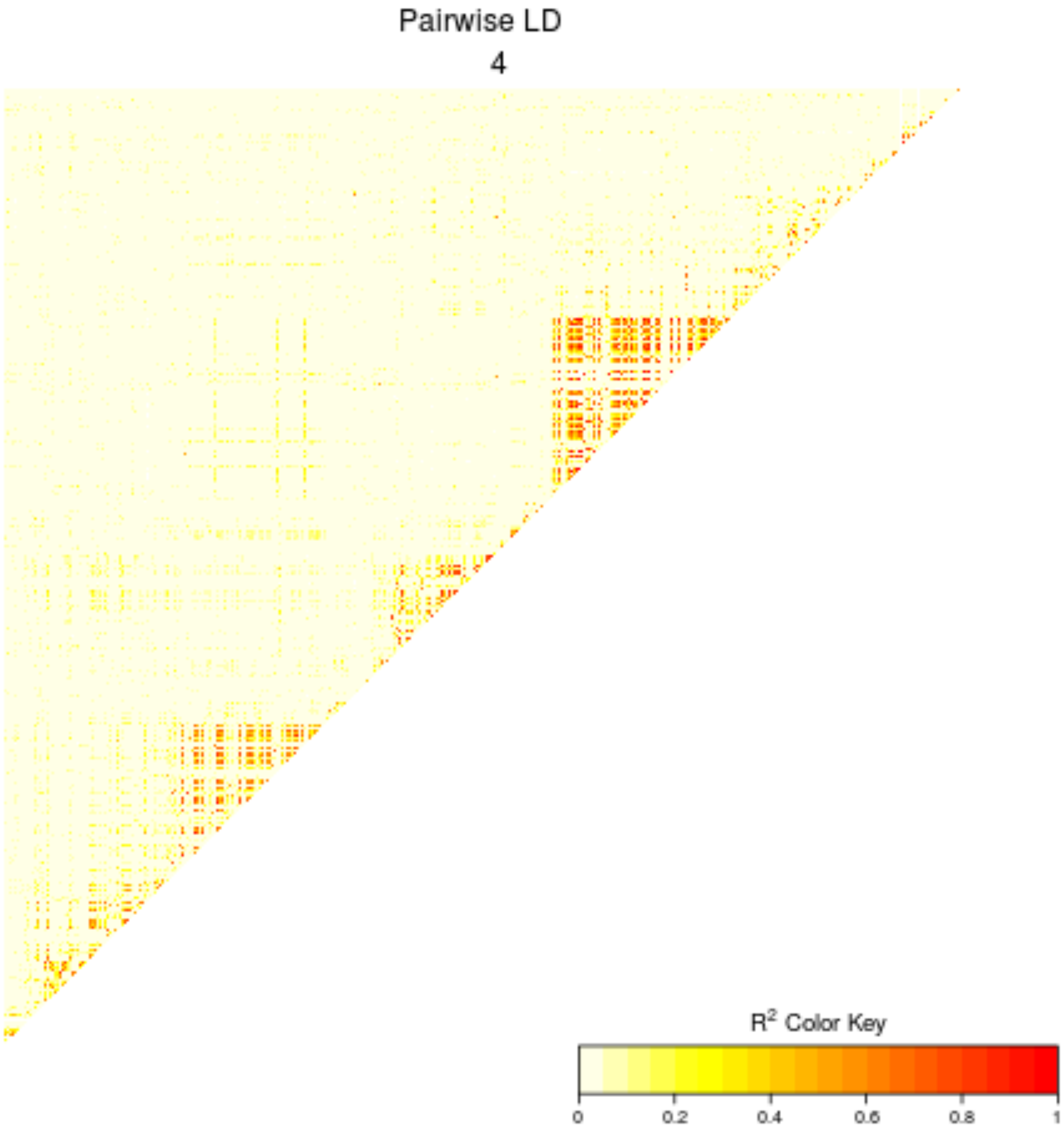
Pairwise LD
4
R2 Color Key
0
0.2
0.4
0.6
0.8
1

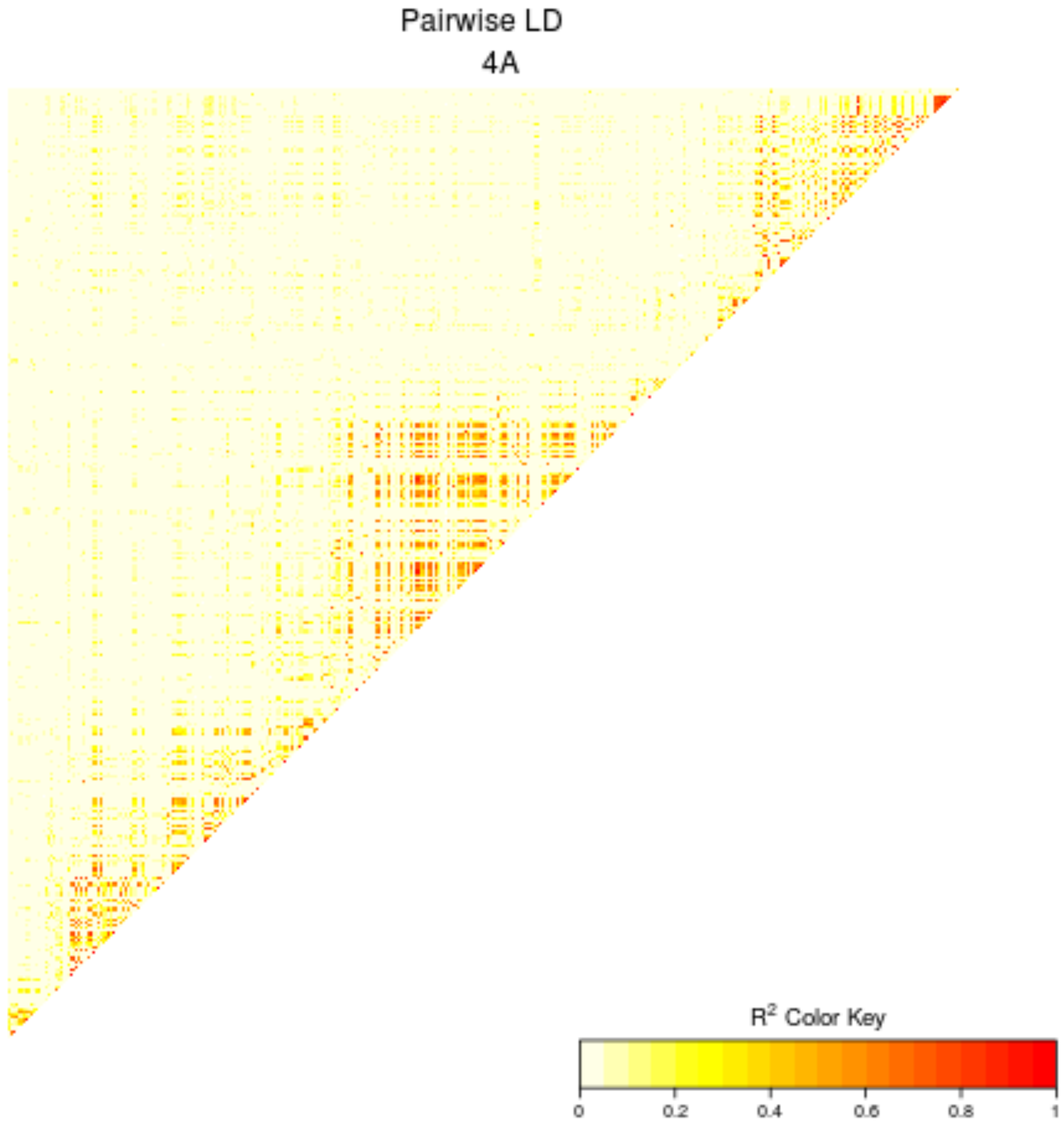

Pairwise LD
4A
R^2 Color Key
0
0.2
0.4
0.6
0.8
1

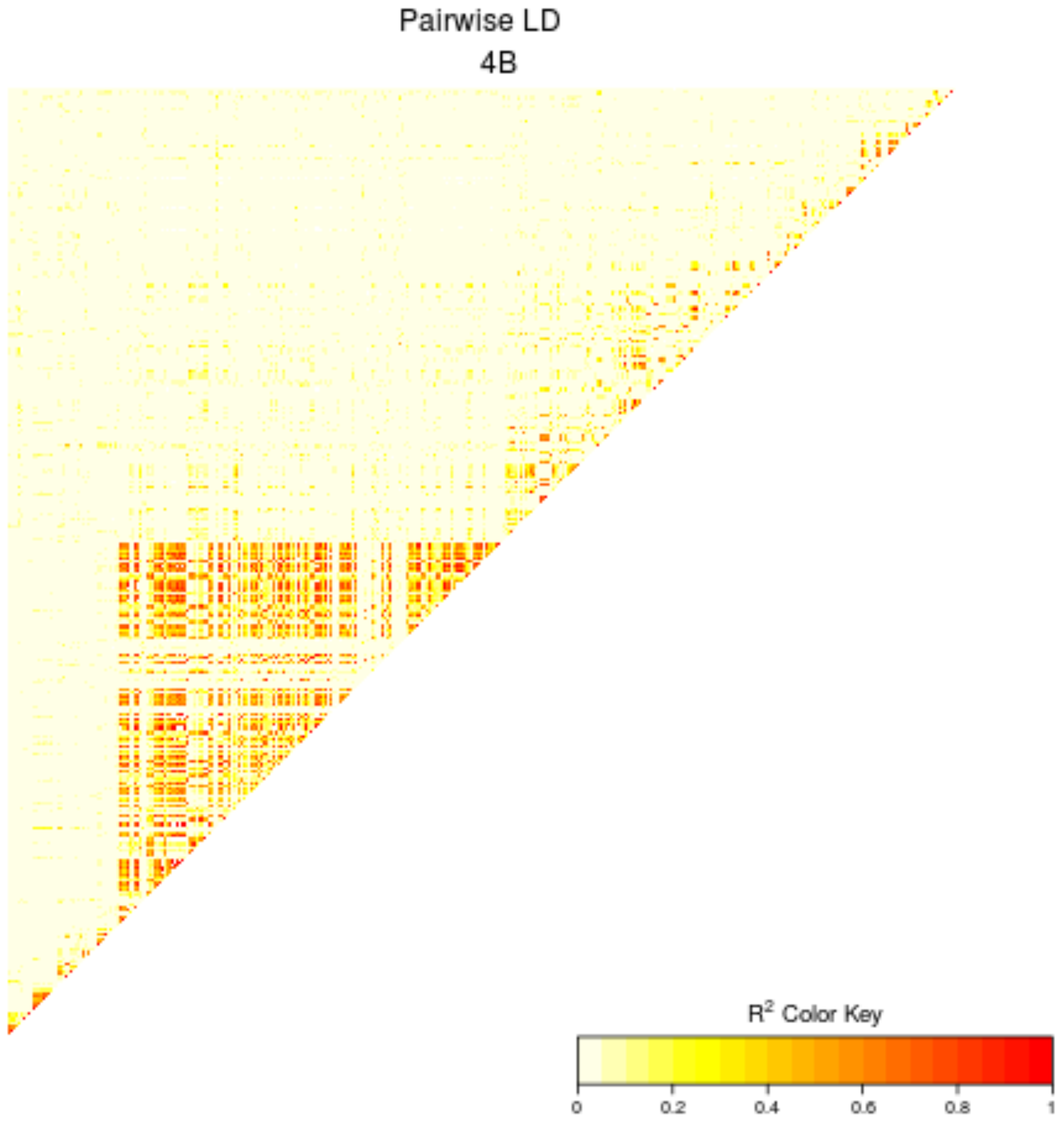
Pairwise LD
4B
R^2 Color Key
0
0.2
0.4
0.6
0.8
1

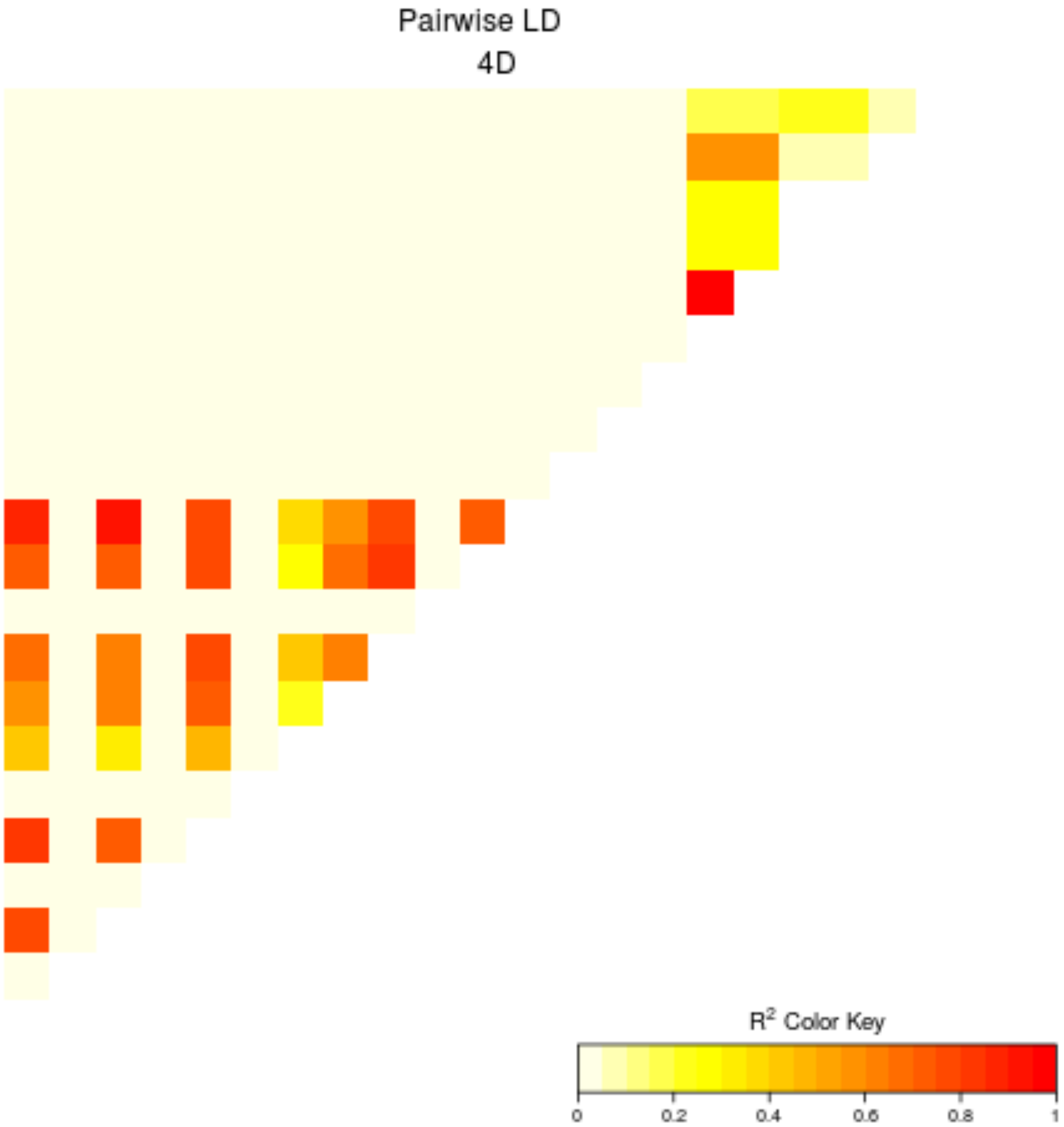
Pairwise LD
4D
R^2 Color Key
0
0.2
0.4
0.6
0.8
1

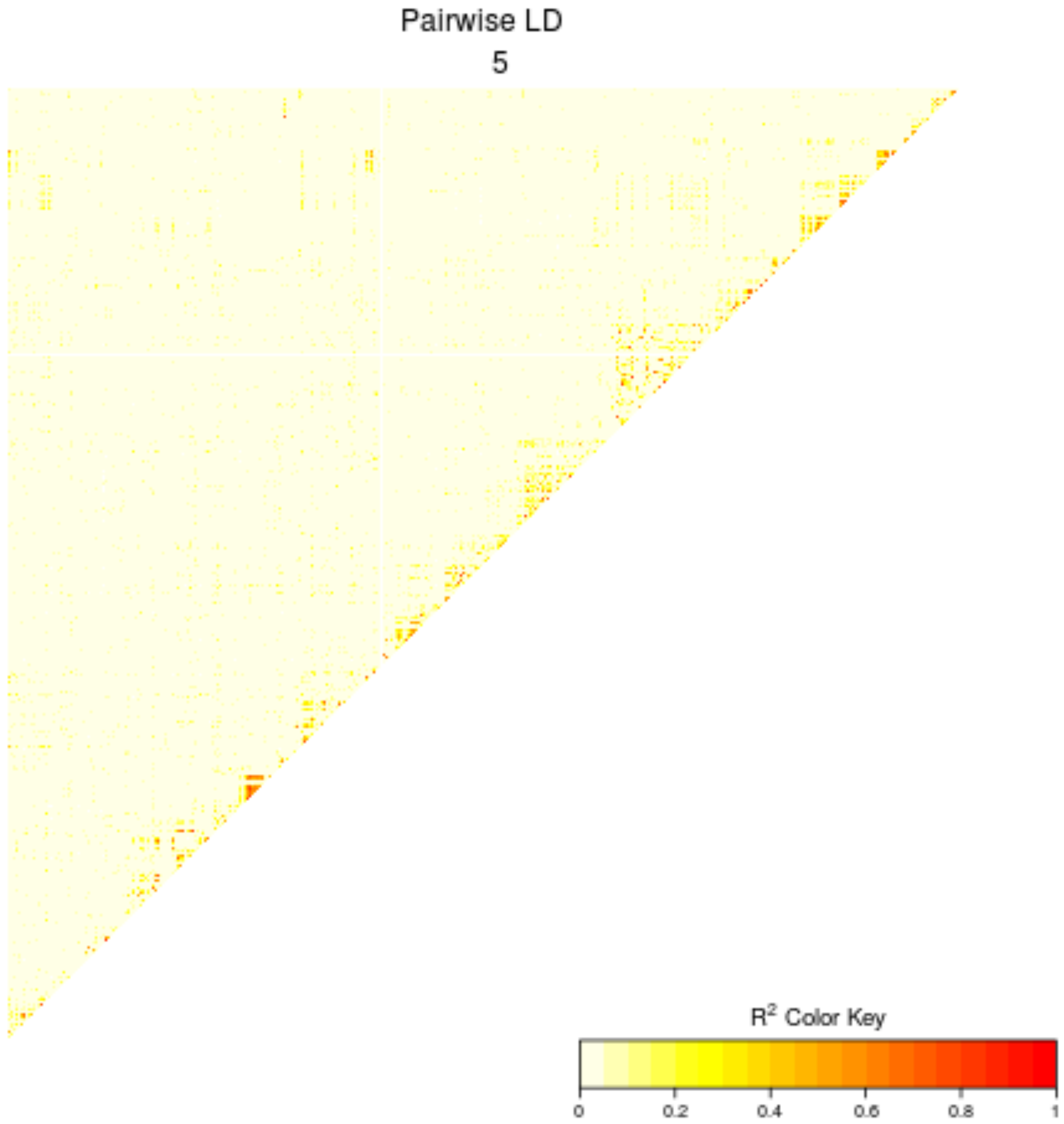

Pairwise LD
5
R2 Color Key
0
0.2
0.4
0.6
0.8
1

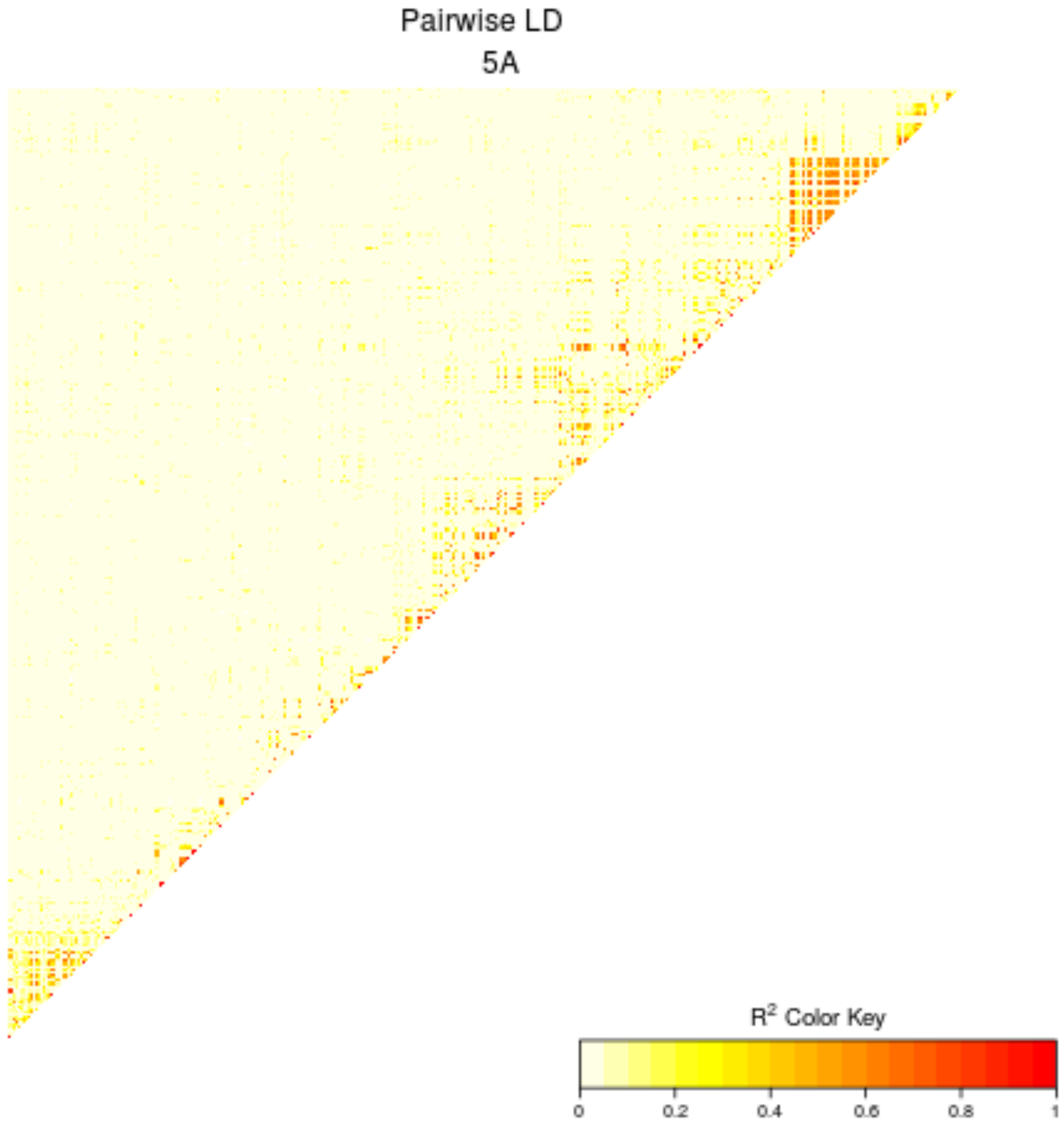
Pairwise LD
5A
R² Color Key
0
0.2
0.4
0.6
0.8
1

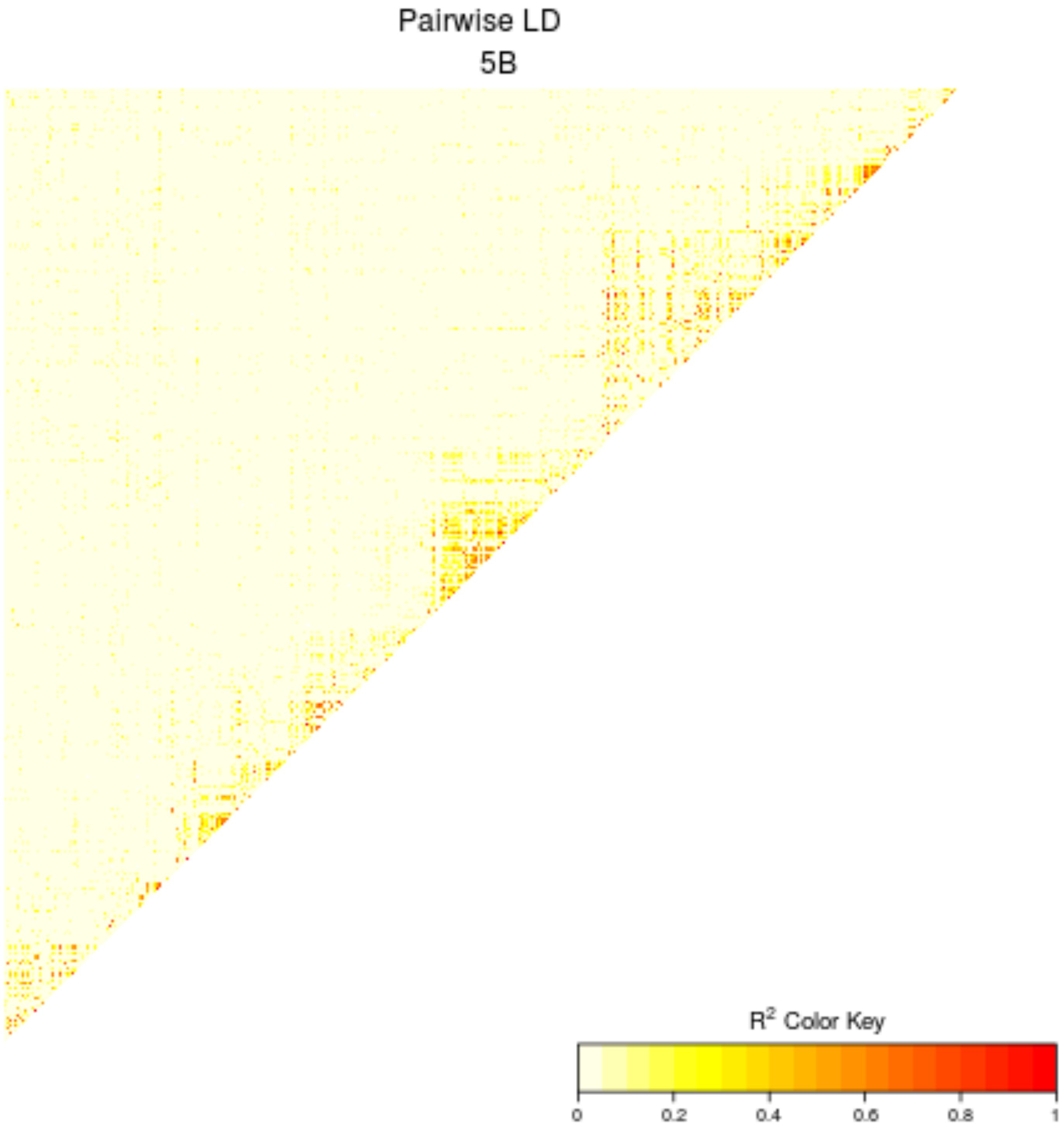
Pairwise LD
5B
R2 Color Key
0
0.2
0.4
0.6
0.8
1

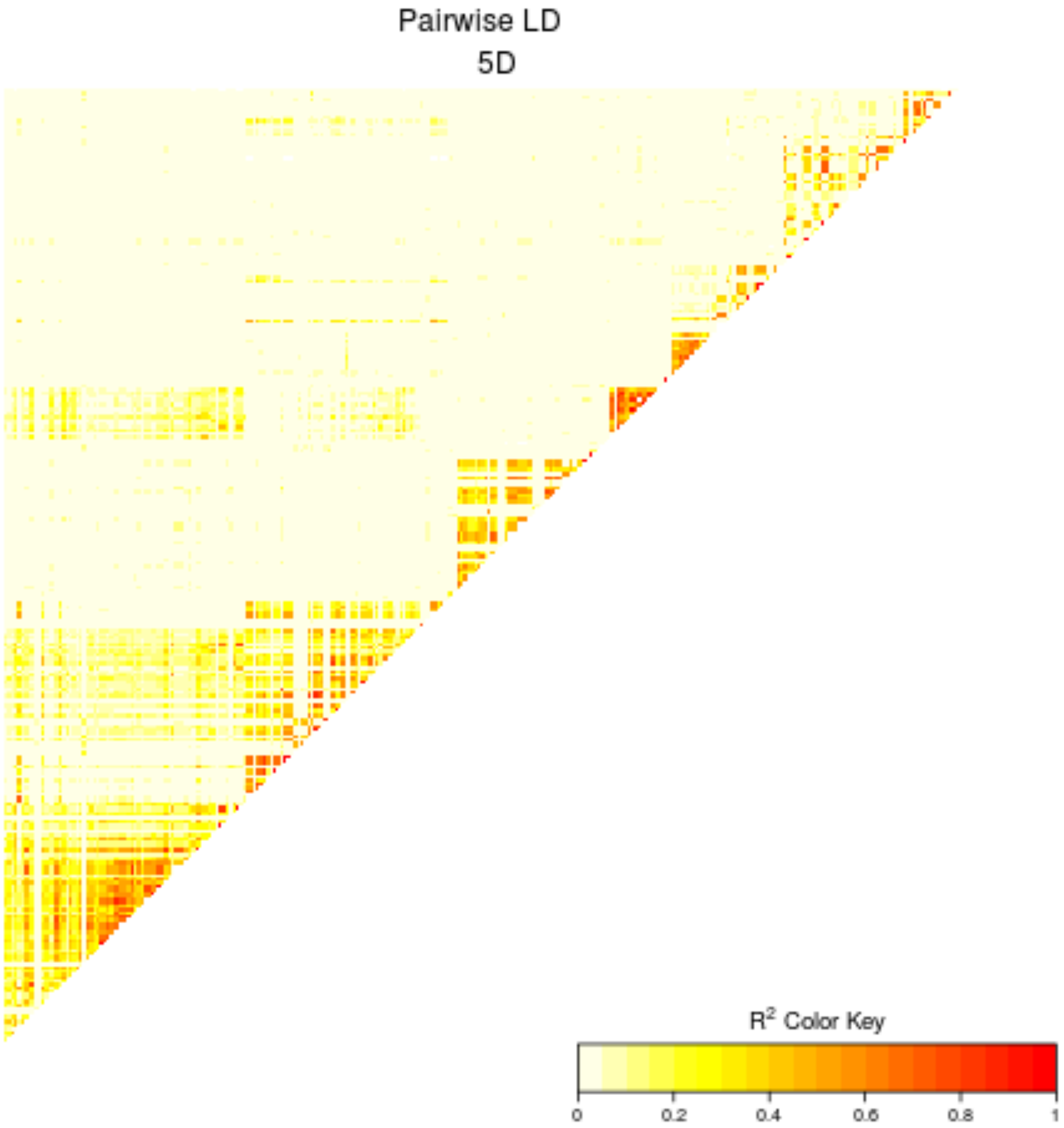
Pairwise LD
5D
R2 Color Key
0
0.2
0.4
0.6
0.8
1

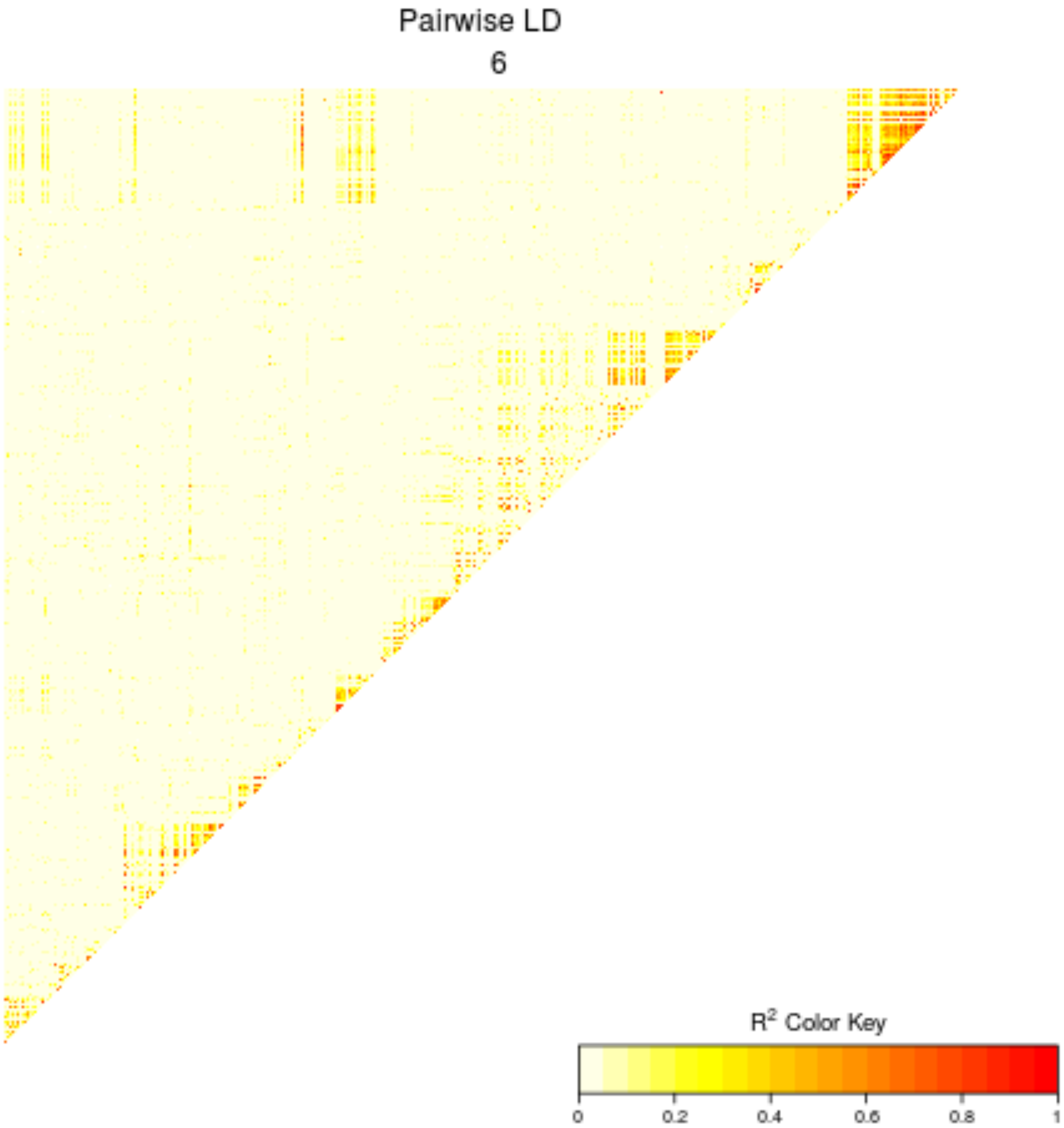
Pairwise LD
6
R2 Color Key
0
0.2
0.4
0.6
0.8
1

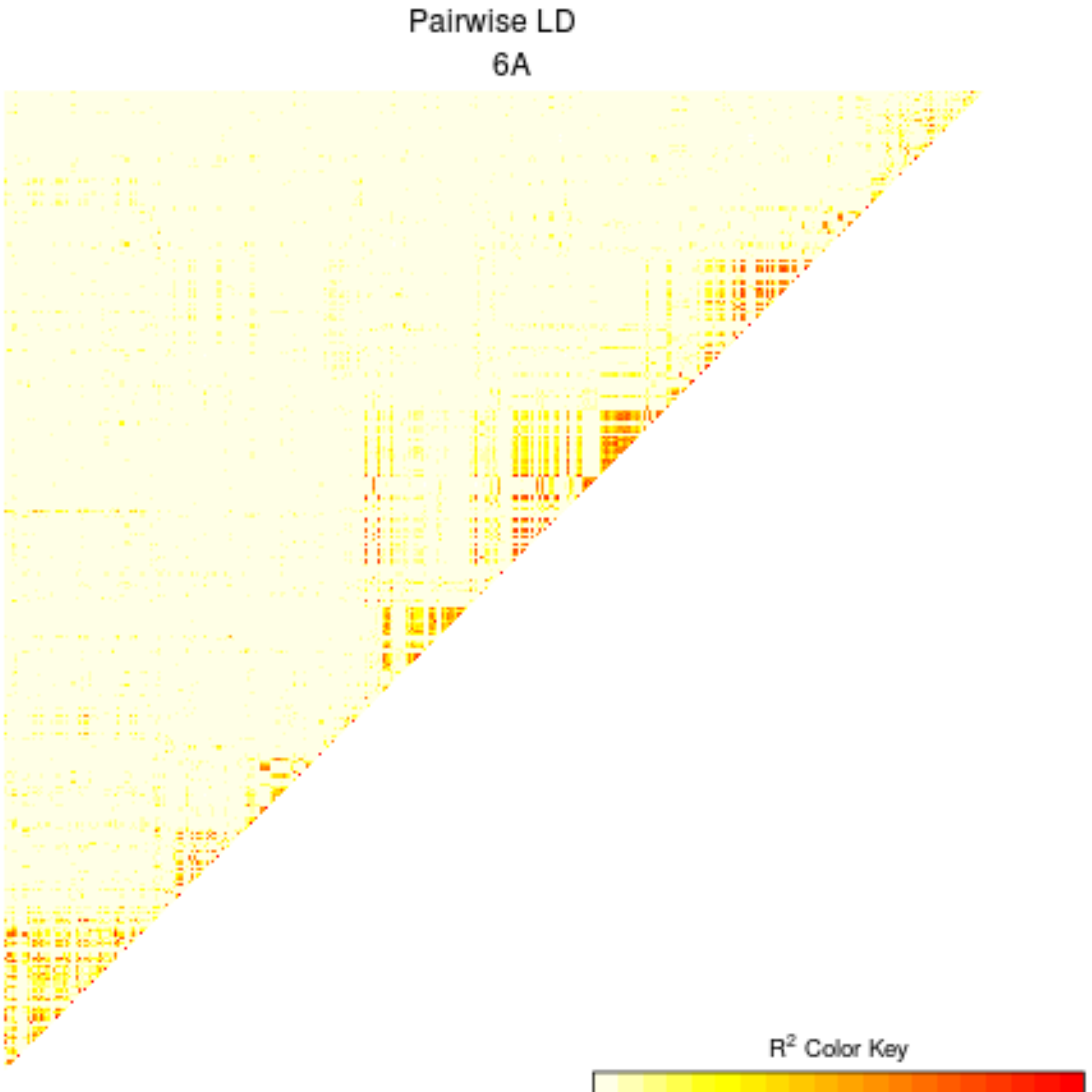
Pairwise LD
6A
R² Color Key
0
0.2
0.4
0.6
0.8
1

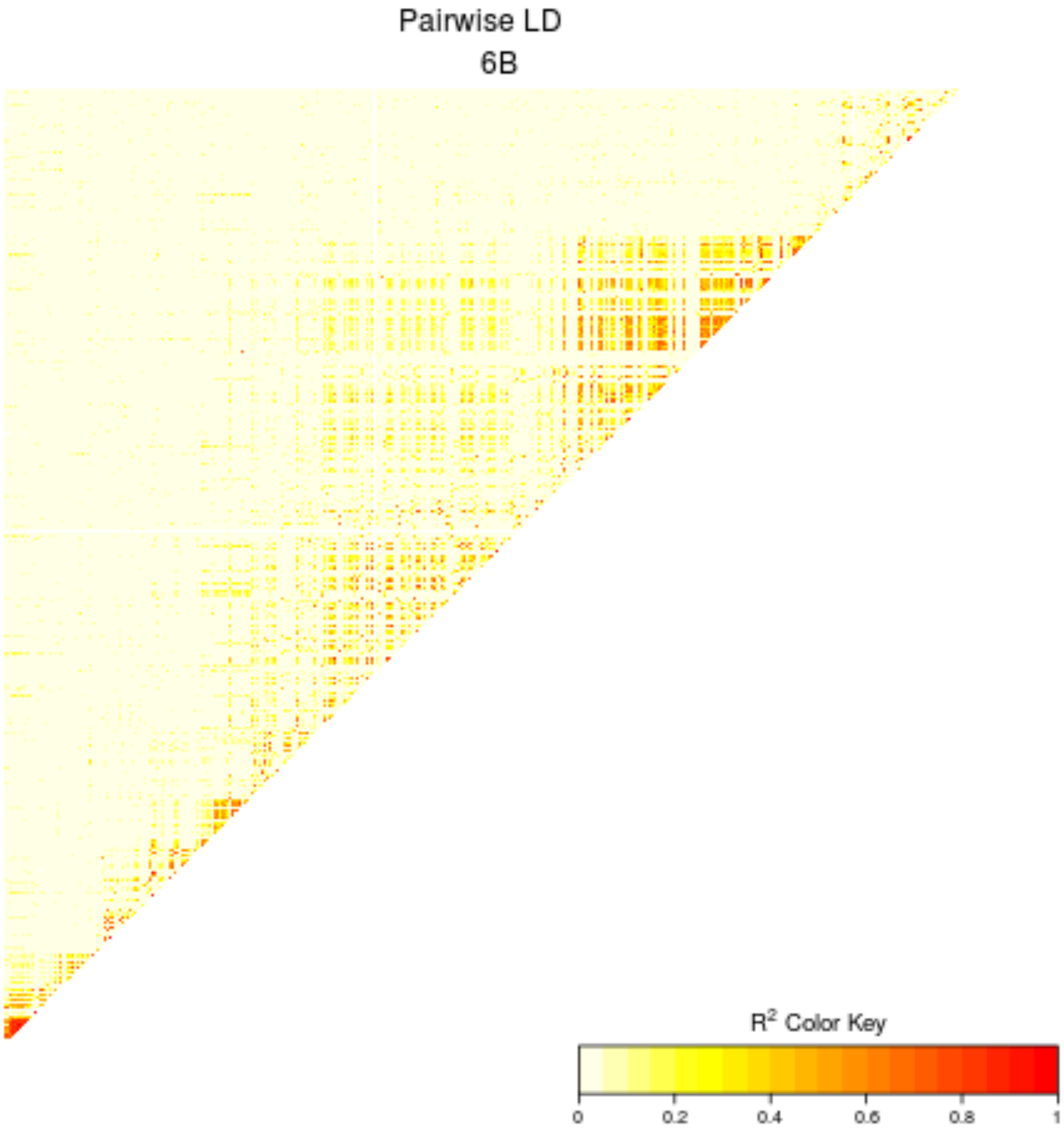
Pairwise LD
6B
R^2 Color Key
0
0.2
0.4
0.6
0.8
1

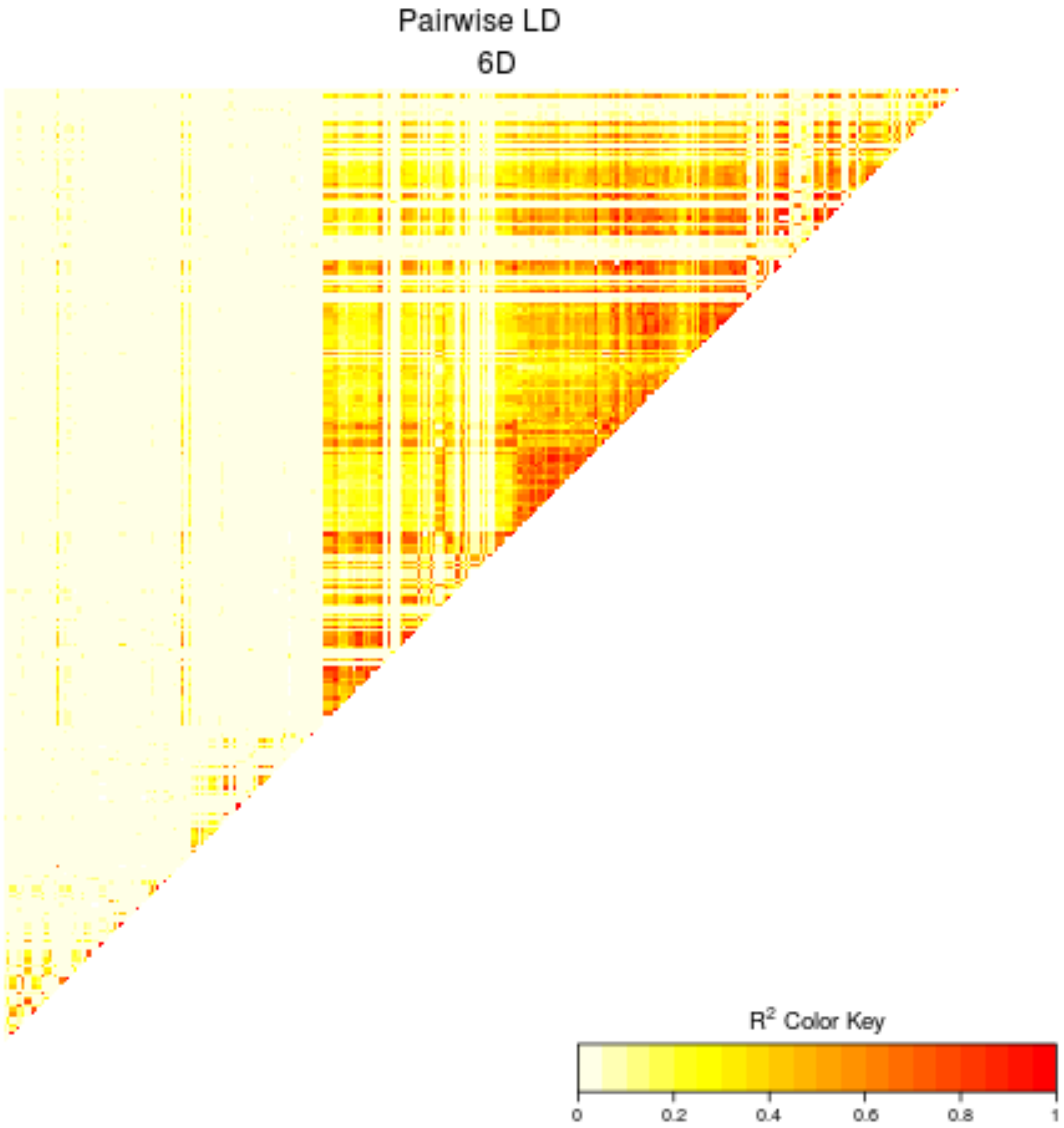
Pairwise LD
6D
R2 Color Key
0
0.2
0.4
0.6
0.8
1

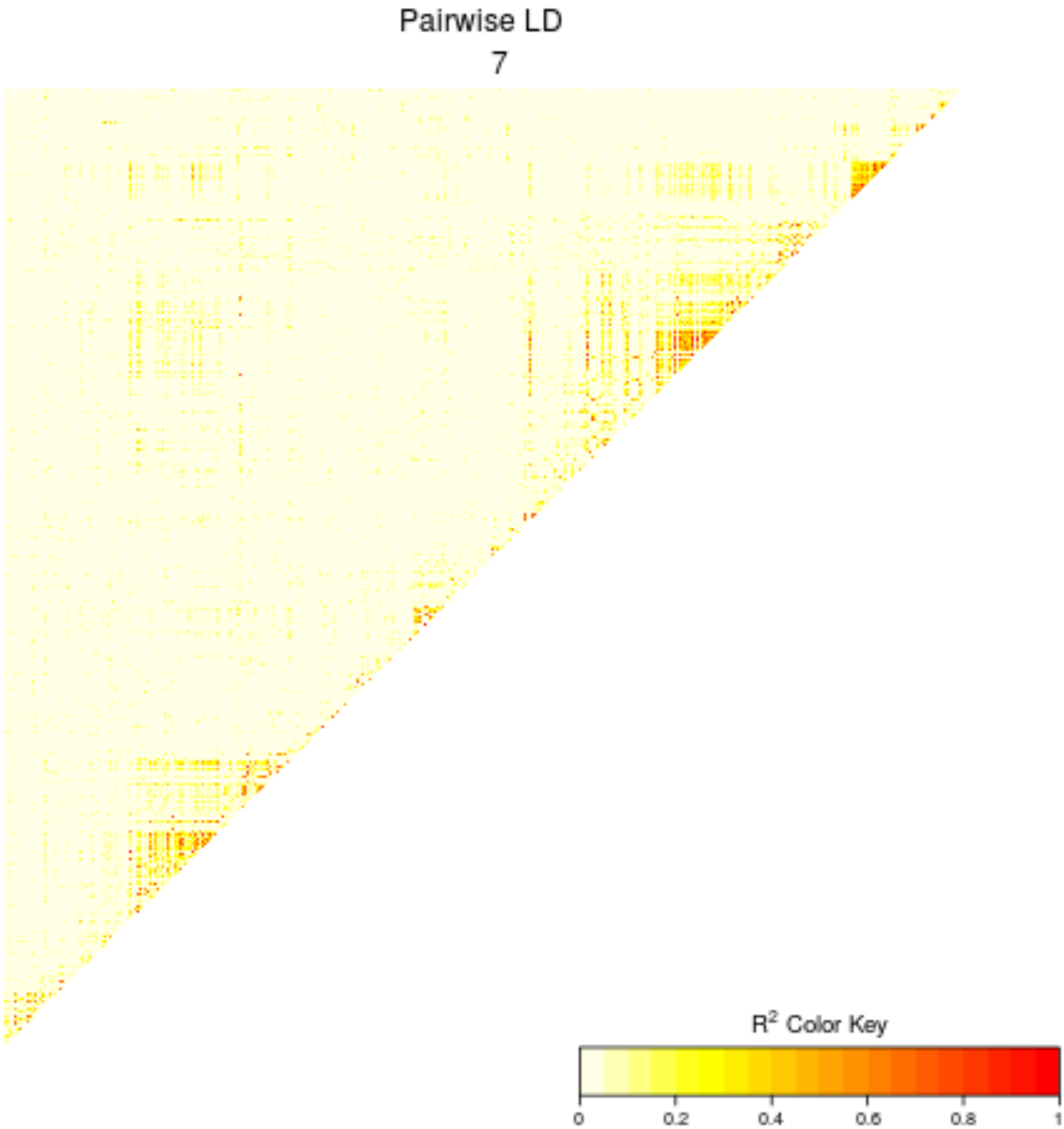
Pairwise LD
7
R^2 Color Key
0
0.2
0.4
0.6
0.8
1

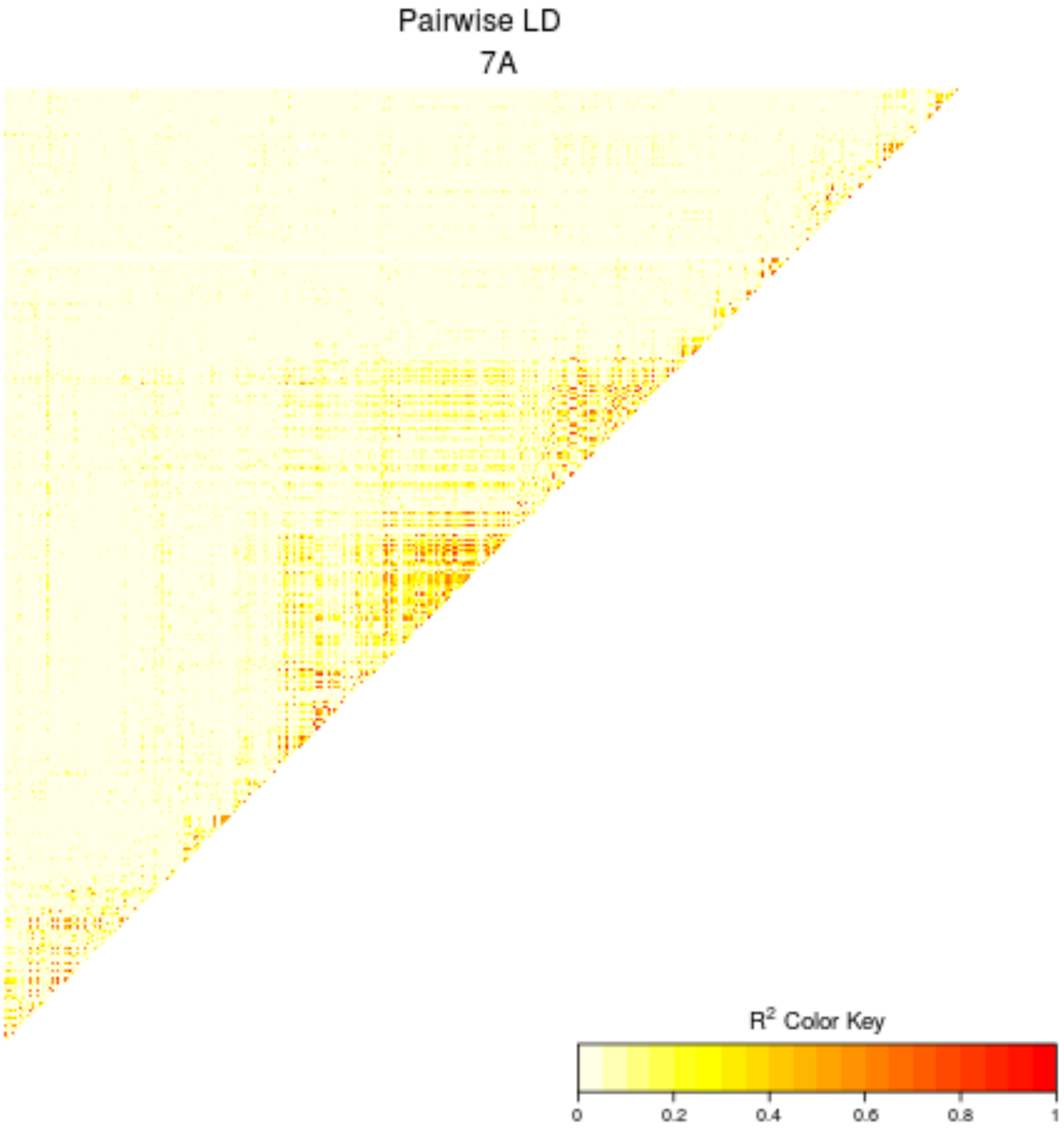
Pairwise LD
7A
R2 Color Key
0
0.2
0.4
0.6
0.8
1

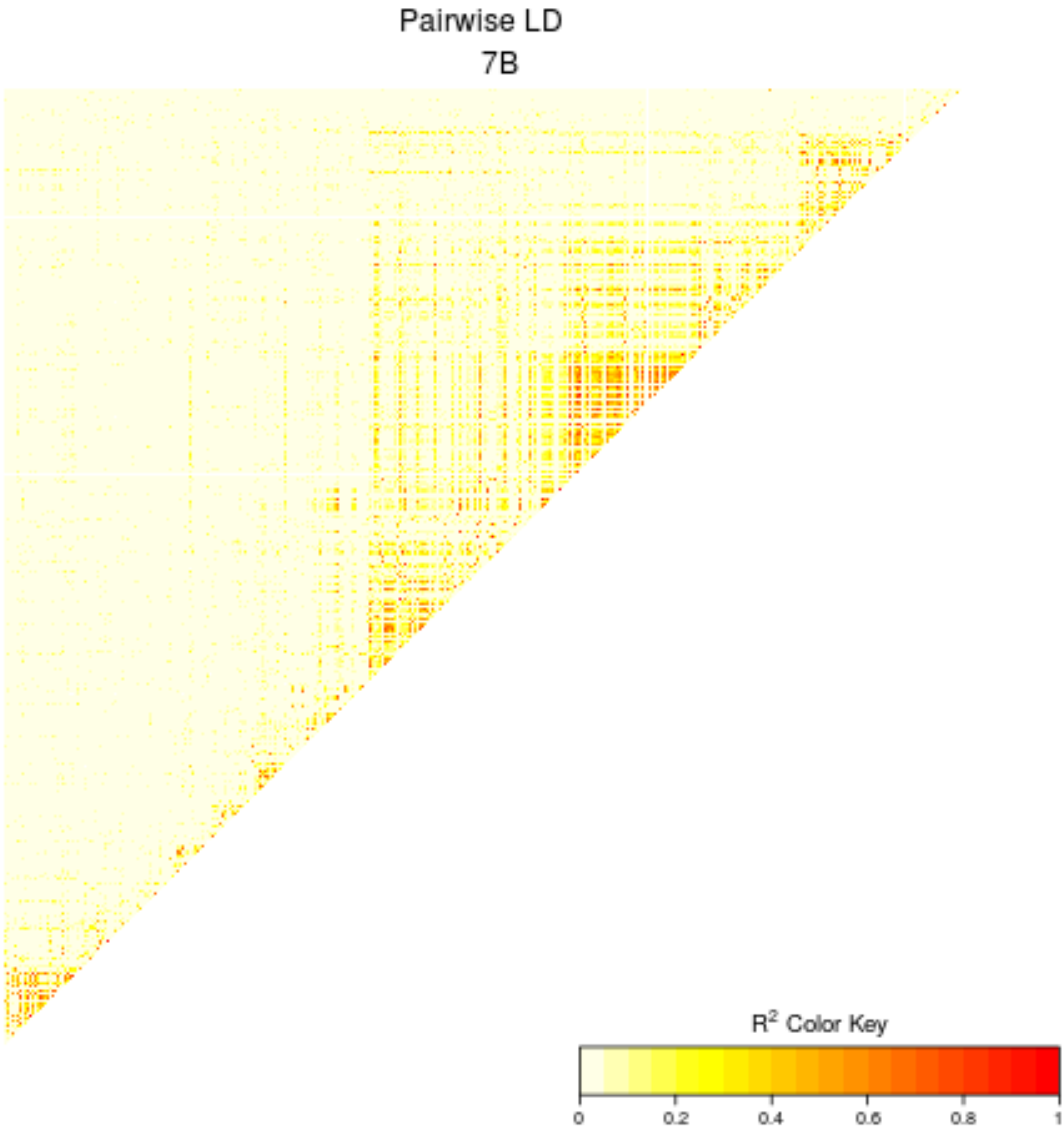
Pairwise LD
7B
R2 Color Key
0
0.2
0.4
0.6
0.8
1

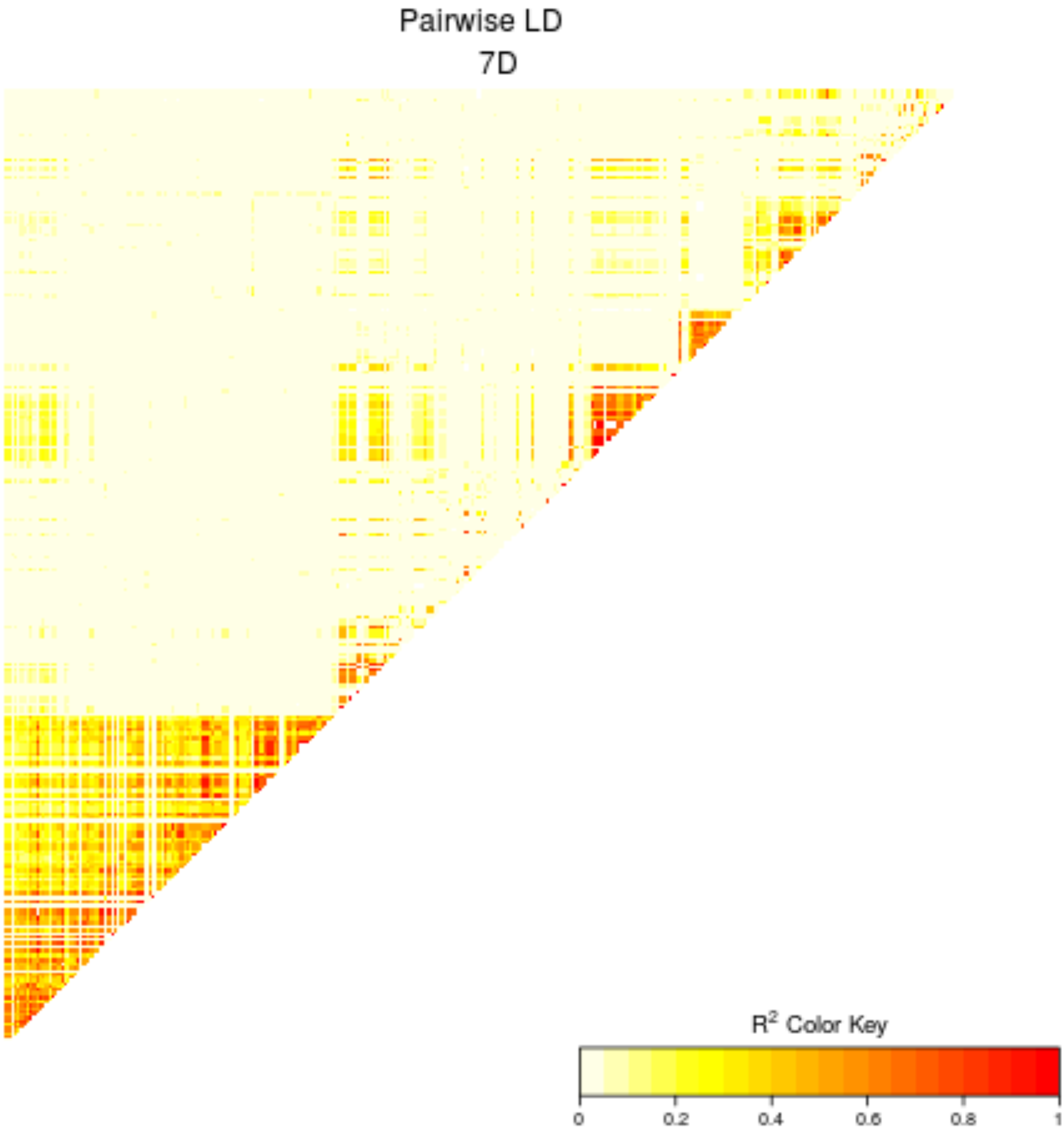

Pairwise LD
7D
R2 Color Key
0
0.2
0.4
0.6
0.8
1
